# NK Cell-Mediated Regulation of Protective Memory Responses against Intracellular Ehrlichial Pathogens

**DOI:** 10.1371/journal.pone.0153223

**Published:** 2016-04-19

**Authors:** Samar Habib, Abdeljabar El Andaloussi, Ahmed Hisham, Nahed Ismail

**Affiliations:** 1 Department of Pathology, University of Pittsburgh, Pittsburgh, Pennsylvania, United States of America; 2 Department of Obstetrics and Gynecology, Medical College of Georgia, Augusta University, Augusta, Georgia, United States of America; Kansas State University, UNITED STATES

## Abstract

*Ehrlichiae* are gram-negative obligate intracellular bacteria that cause potentially fatal human monocytic ehrlichiosis. We previously showed that natural killer (NK) cells play a critical role in host defense against *Ehrlichia* during primary infection. However, the contribution of NK cells to the memory response against *Ehrlichia* remains elusive. Primary infection of C57BL/6 mice with *Ehrlichia muris* provides long-term protection against a second challenge with the highly virulent *Ixodes ovatus Ehrlichia* (IOE), which ordinarily causes fatal disease in naïve mice. Here, we show that the depletion of NK cells in *E*. *muris*-primed mice abrogates the protective memory response against IOE. Approximately, 80% of NK cell-depleted *E*. *muris*-primed mice succumbed to lethal IOE infection on days 8–10 after IOE infection, similar to naïve mice infected with the same dose of IOE. The lack of a recall response in NK cell-depleted mice correlated with an increased bacterial burden, extensive liver injury, decreased frequency of *Ehrlichia*-specific IFN-γ-producing memory CD4^+^ and CD8^+^ T-cells, and a low titer of *Ehrlichia*-specific antibodies. Intraperitoneal infection of mice with *E*. *muris* resulted in the production of IL-15, IL-12, and IFN-γ as well as an expansion of activated NKG2D^+^ NK cells. The adoptive transfer of purified *E*. *muris*-primed hepatic and splenic NK cells into *Rag2*^*-/-*^*Il2rg*^*-/-*^ recipient mice provided protective immunity against challenge with *E*. *muris*. Together, these data suggest that *E*. *muris*-induced memory-like NK cells, which contribute to the protective, recall response against *Ehrlichia*.

## Introduction

Human monocytic ehrlichiosis is an emerging, potentially fatal, tick-borne infectious disease caused by *Ehrlichia chaffeensis*, an obligate intracellular gram-negative bacterium [1–3]. This bacterium primarily infects monocytes and macrophages and causes systemic infection of the reticuloendothelial system [3–5]. The main site of infection and pathology in ehrlichiosis is the liver. We and other investigators have shown that the development of fatal monocytic ehrlichiosis is due to the suppression of protective immunity mediated by IFN-γ and CD4^+^ Th1 cells and excessive inflammation, which leads to immunopathology and multi-organ failure [6–11]. Currently, a broadly effective licensed vaccine is unavailable.

Natural killer (NK) cells are innate lymphocytes that provide rapid and robust host defense against infected cells, including NK cell-mediated antibody-dependent cellular cytotoxicity (ADCC) [12–14]. NK cells can also reciprocally interact with dendritic cells and B cells as well as regulate adaptive T cell-mediated and humoral immunity [15,16]. Recent data have demonstrated that NK cells share several features of the adaptive memory immune response, including their ability to promptly respond to a second challenge with the same antigen they initially encountered (16–24). Accumulating evidence shows that at least some subsets of NK cells respond to certain antigens (viruses or haptens) in a manner that has the hallmarks of adaptive immunity, namely specificity, longevity, and a recall response [13,16,17]. Notably, NK cells with memory function are more confined to the liver.

In this study, we examined the contribution of NK cells to the generation of memory T and B cell responses against *Ehrlichia*. In particular, we investigated whether NK cells acquire a memory-like phenotype following *E*. *muris* infection including their ability to provide recall response against homologous *Ehrlichia* infection. Our data provide the first demonstration that NK cells acquire a memory-like phenotype and mediate a protective recall response against *Ehrlichia* directly and indirectly via the regulation of memory T cell responses during *Ehrlichia* re-challenge. The data obtained in this study will enhance our understanding of the different cellular mechanisms that contribute to the development of an effective and optimal memory response within peripheral organs during infection with intracellular *Ehrlichia*, which is important for vaccine development.

## Materials and Methods

### Ethics Statement

This study was carried out in strict accordance with the recommendation in the guide for the Care and Use of Laboratory Animals of the National Institute of Health (NIH). The animal protocol was approved by the University of Pittsburgh Animal Care and Use Committee (Permit number 14125020). All efforts were made to minimize animal suffering. Mice had access to food and water ad libitum. Infected animals were observed two times/per day, in the morning and in the evening. The body weights and body temperatures were measured. There was no unexpected death. In the survival study, the humane endpoints, at which mice were euthanized, were set as excessive weight loss (>30%), decreased body temperature (drop to <32 degrees), and development of other signs of distress (i.e. decreased movement, lethargy, and having ruffled hair). At that endpoint, mice were humanely euthanized by cervical dislocation. No analgesics and anethethetics were used.

### Mice and bacterial infection

Female C57BL/6J mice (8–12 weeks of age) and *Rag2*^−/−^*Il2rg*^−/−^ mice were obtained from Jackson Laboratories (Bar Harbor, ME, USA). All animals were housed under specific pathogen-free conditions at the Animal Research Facility, University of Pittsburgh. Two species of monocytic *Ehrlichia* were used in this study: the highly virulent IOE, and the mildly virulent *E*. *muris*. IOE and *E*. *muris* stocks were propagated by passage through wild-type C57BL/6 mice. Single-cell suspensions from spleens harvested from mice 7 days post infection (DPI.) were stored in sucrose and potassium phosphate (SPK) buffer (0.5 M K_2_HPO_4_, 0.5 M KH_2_PO_4_, and 0.38 M sucrose) in liquid nitrogen and used as stocks. Mice were infected intraperitoneally (IP) with a lethal high dose of IOE (10^4^ organisms/mouse) or a high dose of *E*. *muris* (2 X 10^5^ organisms/mouse). Mice were then monitored daily for signs of illness and survival.

### *In vivo* NK depletion

NK cells were depleted from *E*. *muris*-primed mice using a non-activating polyclonal antibody (Ab) against asialo-GM1 (Wako Chemicals USA, Inc.). Infected C57BL/6 mice were IP injected with anti-asialo GM1 mAb (25 ug /mouse) or normal rabbit IgG isotype control mAb on days 22, 23, 25 and 26 after primary *E*. *muris* infection. Results from flow cytometry analysis indicated that antibody depletion resulted in a ~95% depletion of NK cells in the spleens and livers of primed mice.

### Isolation of hepatic and splenic NK cells

Spleen and liver tissues were cut into small pieces with a sterile scalpel and passed through 40-μm mesh filters. Single-cell suspensions of splenocytes were prepared as previously described [6,18]. Liver mononuclear cells (LMNCs) were enriched by density-gradient centrifugation as previously described [19–21]. Murine NK cells were isolated from splenocytes and LMNCs by negative selection using the MACS NK cell isolation kit II (Miltenyi Biotec). The purity of unlabeled NK cells was ~85%, as determined by flow cytometry. The activation status of NK cells was not affected by the negative selection process. Since the transferred cells contained ~15% cells other than NK cells, we depleted contaminating CD4^+^T cells in recipient *Rag2*^−/−^*Il2rg*^−/−^ mice by injection of anti-CD4 mAb (200 ug/mouse) (GK1.5, Biolegend) on -1, 0, and 3 DPI.

### Flow cytometry analysis

Splenocytes were harvested, counted, and resuspended in fluorescence-activated staining buffer (Dulbecco’s PBS without Mg^2+^ or Ca^2+^ containing 1% heat-inactivated FCS and 0.09% sodium azide) at a concentration of 10^6^ cells/well. FcRs were blocked with a mAb (clone 2.4G2) against the mouse cell surface Ags CD16 and CD32 for 15 min. The following FITC-, PE-, PerCP-Cy5.5–, Alexa Fluor-, and allophycocyanin-conjugated Abs were purchased from BD Biosciences (San Jose, CA, USA) unless otherwise indicated: anti-CD45.2 (clone 69), anti-CD3 (clone 1452C11), anti-CD11c (clone HL3), anti-CD4 (clone RM4-4), anti-CD11b (clone M1/70), anti-NK1.1 (clone PK136), anti-IFN-γ (clone XMG102), anti-CD62L (clone 1A8), anti-CD44 (3/23), and anti-B220 (clone 16-10A1). Isotype control mAbs, including FITC-, PE-, or allophycocyanin-conjugated hamster IgG1 (A19-3), rat IgG1 (R3-34), rat IgG2a (R35-95), mouse IgG2a (X39), mouse IgG2b (MPC-11), mouse IgG1 (X40), and rat IgG2b (A95-1), were purchased from BioLegend (San Diego, CA, USA). For intracellular cytokine staining, the splenocytes were incubated at 37°C for 4 h in complete medium with the addition of BD Golgi Plug (BD Biosciences, San Jose, CA, USA) according to the manufacturer’s instructions. Lymphocyte and granulocyte populations were gated based on forward and side-scatter parameters; and, 50,000–200,000 events were collected using a BD FACSCalibur flow cytometer (BD Systems, San Jose, CA, USA). Data were analyzed using FlowJo software (TreeStar, Ashland, OR, USA).

### Cytokine measurement

The concentrations of IFN-γ, IL-12, IL-15 and IL-10 in the liver mononuclear cells (LMNCs) culture supernatants were determined using a Quantikine enzyme-linked immunosorbent assay (R&D Systems, Minneapolis, MN, USA) according to the manufacturer’s instructions.

### Detection of nitric oxide (NO) production

NO production was determined by analyzing the level of nitrite (NO^2-^) in the splenocyte culture supernatant from different groups of mice using a colorimetric Griess reaction. Samples were briefly mixed with an equal (1:1) volume of Griess reagent (0.1% N-(1-naphthy) ethylene diamine hydrochloride, 1% sulfonamide, and 2.5% H_3_PO_4_) in 96-well plates, and the absorbance at OD540 were measured using an ELISA reader (Molecular Devices, Sunnyvale, CA, USA) with 1–50 μM sodium nitrite dissolved in distilled water as the standard.

### Measurement of bacterial burden by real-time PCR (RT-PCR)

The number of *Ehrlichia* organisms in frozen stocks and the bacterial burden in different organs were measured by quantitative RT-PCR using an iCycler IQ multicolor real-time detection system (Bio-Rad, Hercules, CA, USA), as previously described [22]. The sequences of primer sets used that target both the *E*. *muris* and the IOE *dsb* (a thiol-disulfide oxidoreductase) genes, the eukaryotic housekeeping gene GAPDH, and specific probes have been previously described [6,22,23]. Results were normalized to the expression levels of the GAPDH gene in the same sample and were expressed as copy numbers per 10^4^ GAPDH copies. PCR analyses were considered negative for *Ehrlichia* DNA if the critical threshold values exceeded 40 cycles.

### Histopathology staining of liver sections

Liver segments were fixed in 10% neutral buffered formalin, dehydrated in graded alcohols, and embedded in paraffin wax. Sections (3-mm thick) were collected on coated slides and stained with H&E.

### Measurement of *Ehrlichia*-specific antibodies by immunofluorescence assay (IFA)

Serum samples from infected and control mice were measured for antigen (Ag)- specific IgG Abs through an indirect immunofluorescence assay using *E*. *muris* as an *Ehrlichia* cross-reactive antigen, as previously described [3,6,23–25]. A serial two-fold dilution of serum samples was applied to fixed Ag slides. After incubation at 37°C for 30 min in a humid chamber, slides were stained with FITC-labeled anti-mouse IgG (BD eBioscience) at a dilution of 1/100. The slides were examined under a fluorescence microscope (Nikon, Tokyo, Japan). Serological titers were expressed as the reciprocals of the highest dilution at which specific fluorescence was detected.

### Statistical analysis

All of the data presented are representative of two or three independent experiments that yielded similar results. Data are represented by means and standard deviations (SD). Two groups analysis was performed using an unpaired two-tailed *t* test. For comparison of multiple experimental groups, we used one–way analysis of variance (ANOVA) with Bonferroni’s procedure. To determine whether the difference in survival between different mice groups was significant, data were analyzed by the Breslow-Wilcoxon Test. All statistical analyses were performed using GraphPad Prism (GraphPad Software Inc., La Jolla, CA, USA). Differences with *P* values of 0.05, 0.01, and 0.001 were considered slightly (*), moderately (**), and highly (***) significant, respectively.

## Results

### Primary *E*. *muris* but not IOE infection induces expansion and activation of NK cells

**Fig 1:**
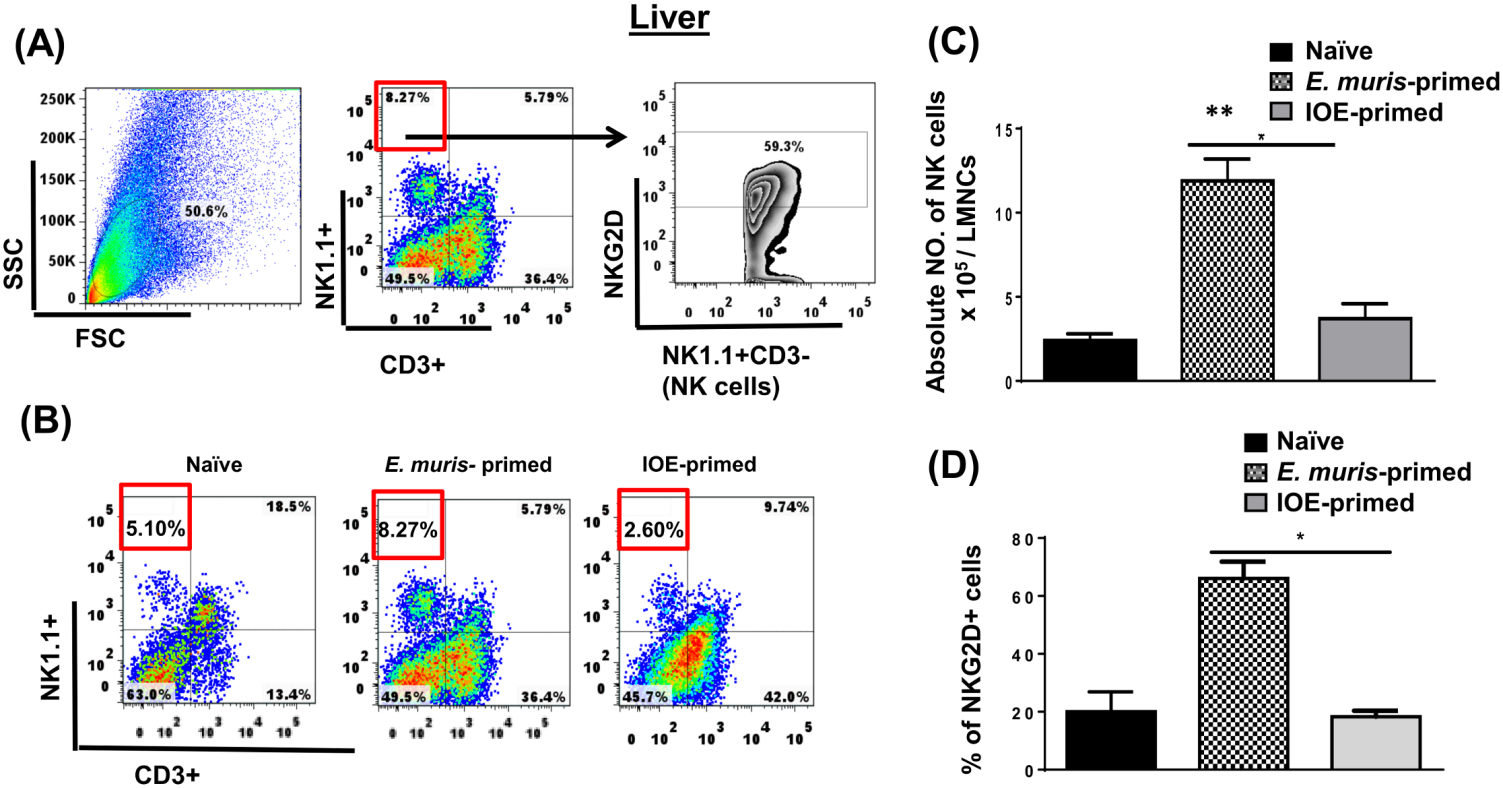
NK cells expand and persist in the liver of *E*. *muris*- but not IOE-primed mice. Liver mononuclear cells were isolated from indicated mice groups on day 21 post-infection, and the frequency and activation of NK cells were analyzed. **(A)** shows gating strategy on NK cells and activation marker NKG2D. **(B)** The percentage and **(C)** absolute number of NK cells in the livers of indicated mice groups. as determined by flow cytometry. **(D)** The percentage of activated NK cells expressing NKG2D. The results demonstrate a higher frequency of activated NKG2D^+^ NK cells in the livers of *E*. *muris*-primed mice as compared to other groups (* *P* <0.05 and ** *P* <0.01). The data shown are the means ± SD from three mice per group and are representative of three independent experiments.

**Fig 2:**
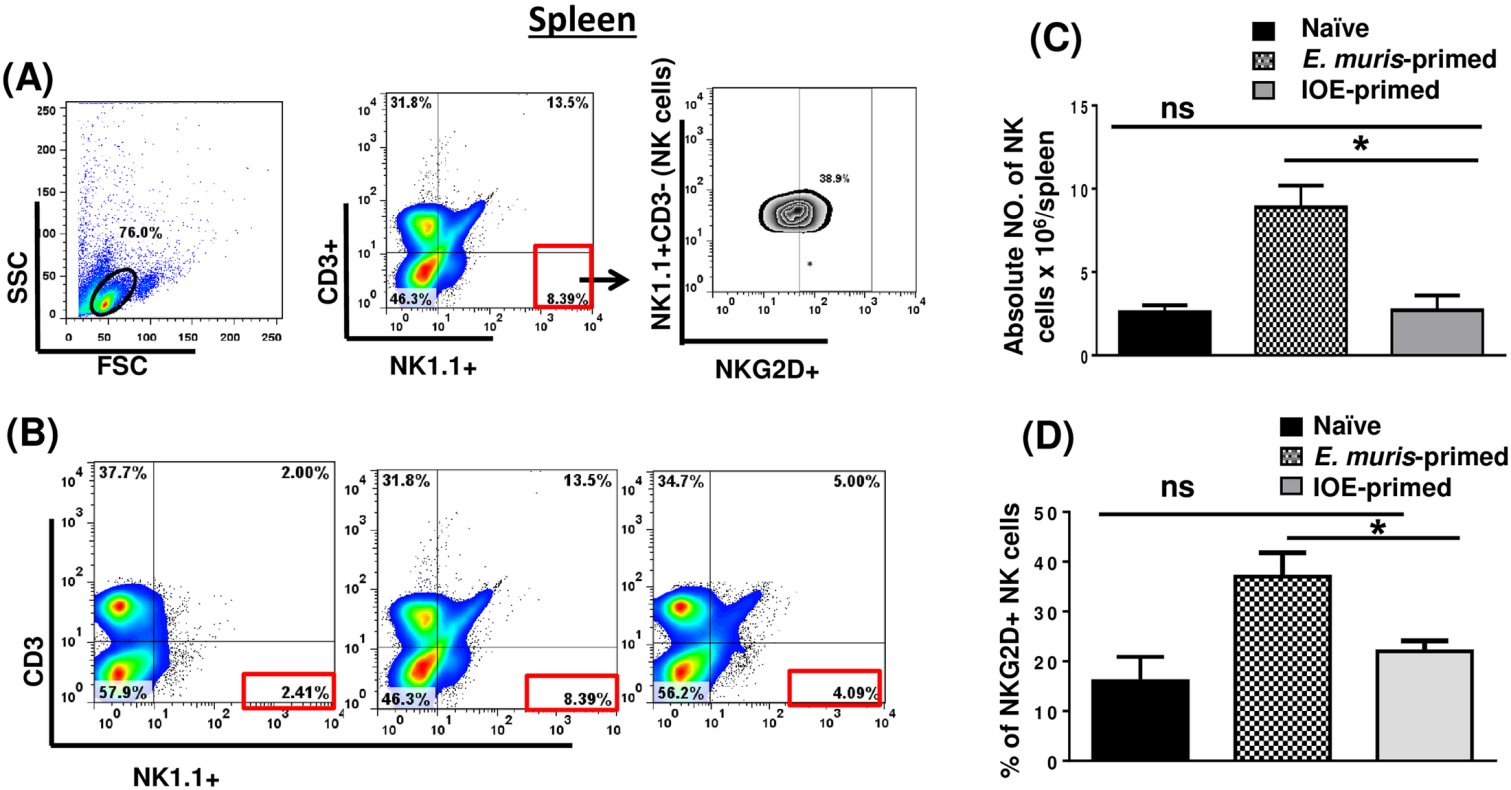
NK cells expand and persist in the spleen of *E*. *muris*- but not IOE-primed mice. Splenocytes were isolated from indicated mice groups on day 21 post-infection, and the frequency and activation of NK cells were analyzed. **(A)** The gating strategy on NK cells and activation marker NKG2D. **(B)** The percentage and **(C)** absolute number of NK cells in the spleens of indicated mice groups as determined by flow cytometry. **(D)** The percentage of activated NK cells expressing NKG2D. The results demonstrate a higher frequency of activated NKG2D^+^ NK cells in the spleens of *E*. *muris*-primed mice as compared to other groups (* *P* <0.05). The data shown are the means ± SD from three mice per group and are representative of three independent experiments.

We previously showed that a primary non-lethal infection of wild type (WT)-B6 mice with *E*. *muris* (EM), but not a sublethal IOE infection, provides long-term protection of primed mice against an ordinarily lethal secondary IOE infection [23,25]. *E*. *muris*, but not IOE, causes persistent infection, which is critical for the induction and maintenance of memory CD4^+^ T cells. However, the adoptive transfer of memory CD4^+^ T cells alone provides partial protection to naïve mice against lethal IOE infection [6]. Thus, we hypothesized that the protective recall response in *E*. *muris*-primed mice could be mediated by cells other than memory CD4^+^ T cells. Because the liver, which is enriched with NK and NKT cells, serves as the main site of infection in ehrlichiosis, we examined whether cross-protection in *E*. *muris-*primed mice was due to differences in NK cell responses. To this end, we analyzed the activation status, and frequency of NK cells in the liver and spleen of mice infected with *E*. *muris* or IOE via the IP route. Our data demonstrated that NK cells expand and persist in the liver Fig 1A–1C of *E*. *muris-*, but not IOE-, primed mice 21 DPI [the time point that corresponds to the contraction of effector T cells and the generation of central and effector memory T cells [23,25]. Our data further showed that approximately 70% of liver NK cells in *E*. *muris*-primed mice expressed NK cell-activating receptor (NKG2D) Fig 1D, suggesting that these cells were activated *in vivo*. Similar to hepatic NK cells, the frequency of splenic NK cells was higher in the spleen of *E*. *muris*-primed mice as compared to IOE-primed mice and uninfected controls Fig 2A–2C. Approximately 40% of *E*. *muris*-primed splenic NK cells expressed NKG2D, while ~ 20% of IOE-primed splenic NK cells expressed NKG2D Fig 2D. Since the total number of splenic NK cells was significantly higher in *E*. *muris*-infected mice than that detected in IOE-infected mice, these data suggest that *E*. *muris* also induces significant expansion of activated NKG2D^+^NK cells in the spleen compared to IOE-primed mice. These data suggest that *E*. *muris* infection promotes the expansion of activated NK cells that persist in the primed host, in the liver and spleen.

### NK cell depletion leads to an impaired protective recall response against *Ehrlichia*

**Fig 3:**
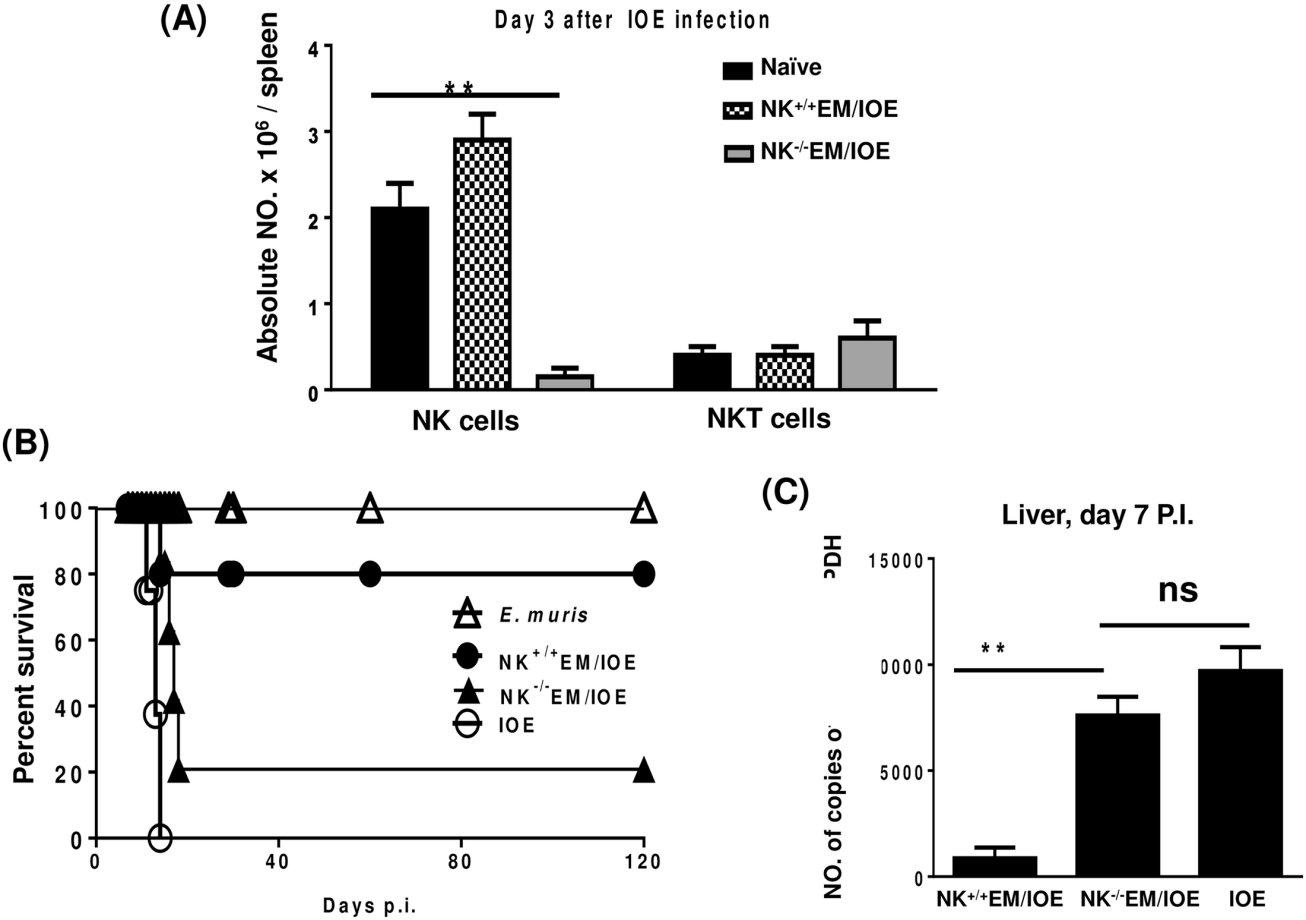
Secondary high-dose challenge with IOE is fatal in NK-depleted EM/IOE-infected mice. C57BL/6 mice were infected with a high dose of *E*. *muris*, and were either depleted of NK cells at 22, 23, 24, and 26 DPI (NK^-/-^ EM/IOE) or treated with isotype control antibody (NK^+/+^EM/IOE). Both groups of mice (n = 12 mice/group) were re-challenged with a high dose of IOE on day 28 after infection. The other control group includes naïve mice infected only with IOE. Depletion of *E*. *muris*-primed mice with anti-asialo GM1 antibodies resulted in: **A)** ~95% depletion of NK cells on day 3 after IOE infection; **B)** decreased survival of NK^-/-^EM/IOE mice group; and **C)** higher bacterial burden in the liver of NK^-/-^EM/IOE mice on day 7 after IOE infection as compared with NK^+/+^EM/IOE-infected mice. The data represent the means ± SD from four mice per group and are representative of three independent experiments.

To further examine the contribution of NK cells to the memory response against *Ehrlichia*, we depleted NK cells from *E*. *muris*-primed mice before we challenged these mice with IOE. The treatment of *E*. *muris*-primed mice with anti-asialo GM1 antibodies did not influence the number of T cells and NKT cells on day 3 S1 Fig or day 5 S2 Fig post-infection, respectively. The treatment of *E*. *muris*-primed mice with anti-asialo GM1 antibodies did not also influence the number of CD11b^+^ macrophages (data not shown). However, anti-asialo GM1 antibodies resulted in a ~95% depletion of NK cells on day 3 after IOE infection Fig 3A.

Consistent with our previous studies [6,23], 80% of the *E*. *muris*-primed mice treated with isotype control antibody (referred to as NK^+/+^EM/IOE) survived re-challenge with an ordinarily lethal dose of IOE up to 120 days after IOE challenge. Notably, the depletion of NK cells in *E*. *muris*-primed mice resulted in the loss of a protective recall response, such that ~80% of NK cell-depleted mice (referred to as NK^-/-^EM/IOE) succumbed to lethal IOE infection between days 15 and 17 post-IOE challenge Fig 3B. The depletion of NK cells in NK^-/-^EM/IOE mice also led to an increased bacterial burden in the liver on day 7 following IOE challenge Fig 3C. These data suggest that NK cells contribute to effective bacterial clearance and protective memory response against *Ehrlichia*.

### Antigen-specific CD4^+^ T cell responses are impaired in NK cell-depleted EM/IOE mice

**Fig 4:**
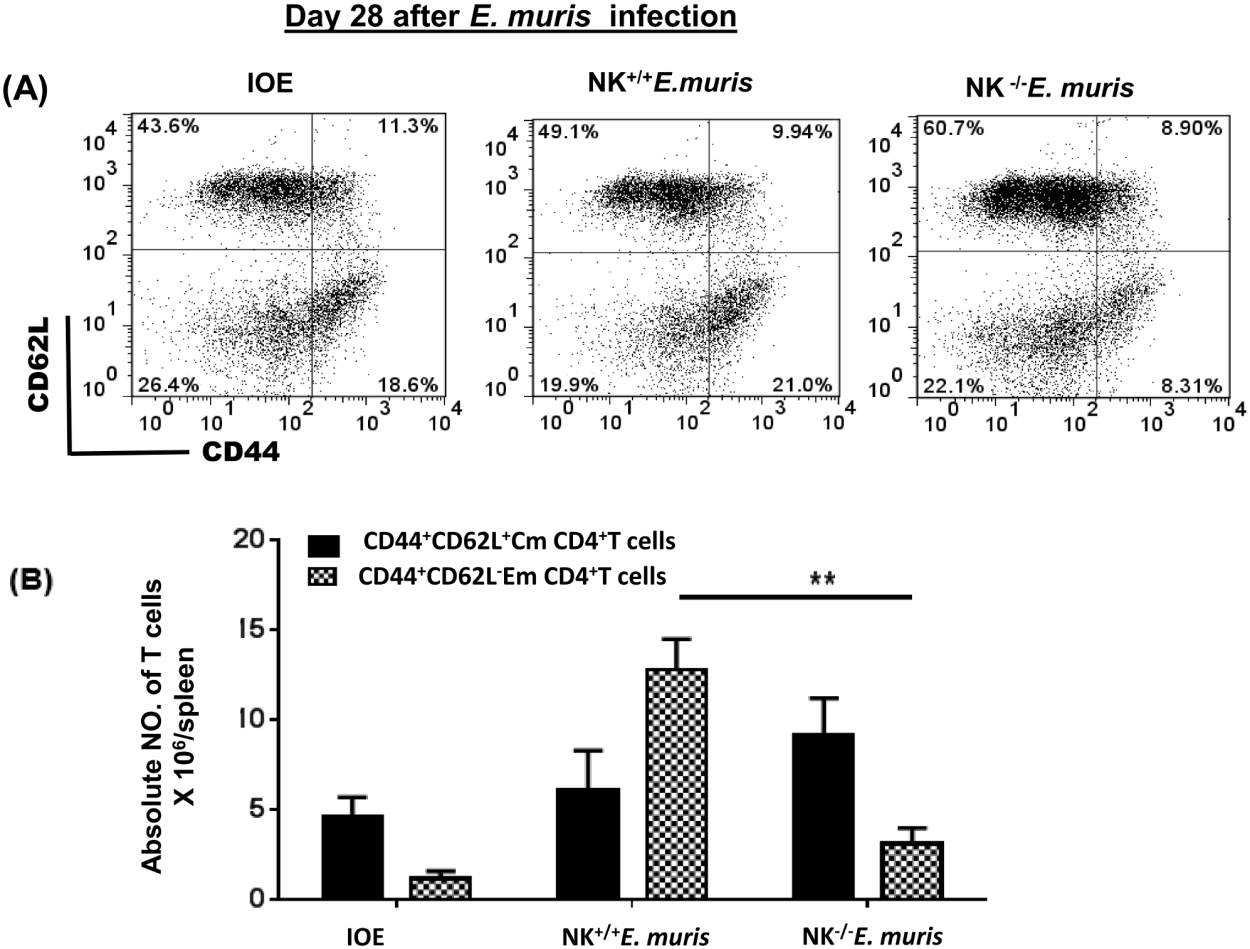
NK cell depletion in *E*. *muris-*primed mice decreases expansion of memory T cells. Splenocytes from different groups of mice were stimulated with *E*. *muris* antigens and the frequency of memory cells was determined by flow cytometry. Lower percentages **(A)** and absolute numbers **(B)** of effector/effector memory (Em) (CD44^high^ CD62L^low^), but not central memory (Cm) (CD44^high^ CD62L^high^), CD4^+^ T cells in the spleens of NK^-/-^*E*. *muris* compared with those found in NK^+/+^*E*. *muris* or naïve mice infected with IOE on day 28 post-infection. ** indicate *P* < 0.01. Data are presented as the means ± SD of four mice per group from two independent experiments. ** indicates *P* <0.01.

To further examine the effect of NK cell depletion on the protective memory response, we measured the frequency and function of memory CD4^+^ T cells. Splenocytes were harvested from all groups of mice 28 DPI, and stimulated with *E*. *muris* antigens (Ags). Our data show that the spleens of NK^-/-^EM/IOE mice had a significantly lower percentage Fig 4A and absolute number Fig 4B of Ag-specific CD44^+^CD62L^-^ effector memory (Em) CD4^+^ T cells 28 DPI after *E*. *muris* infection as compared with the number of Em CD4^+^ T cells in sham (isotype) control *E*. *muris*-infected mice.

**Fig 5:**
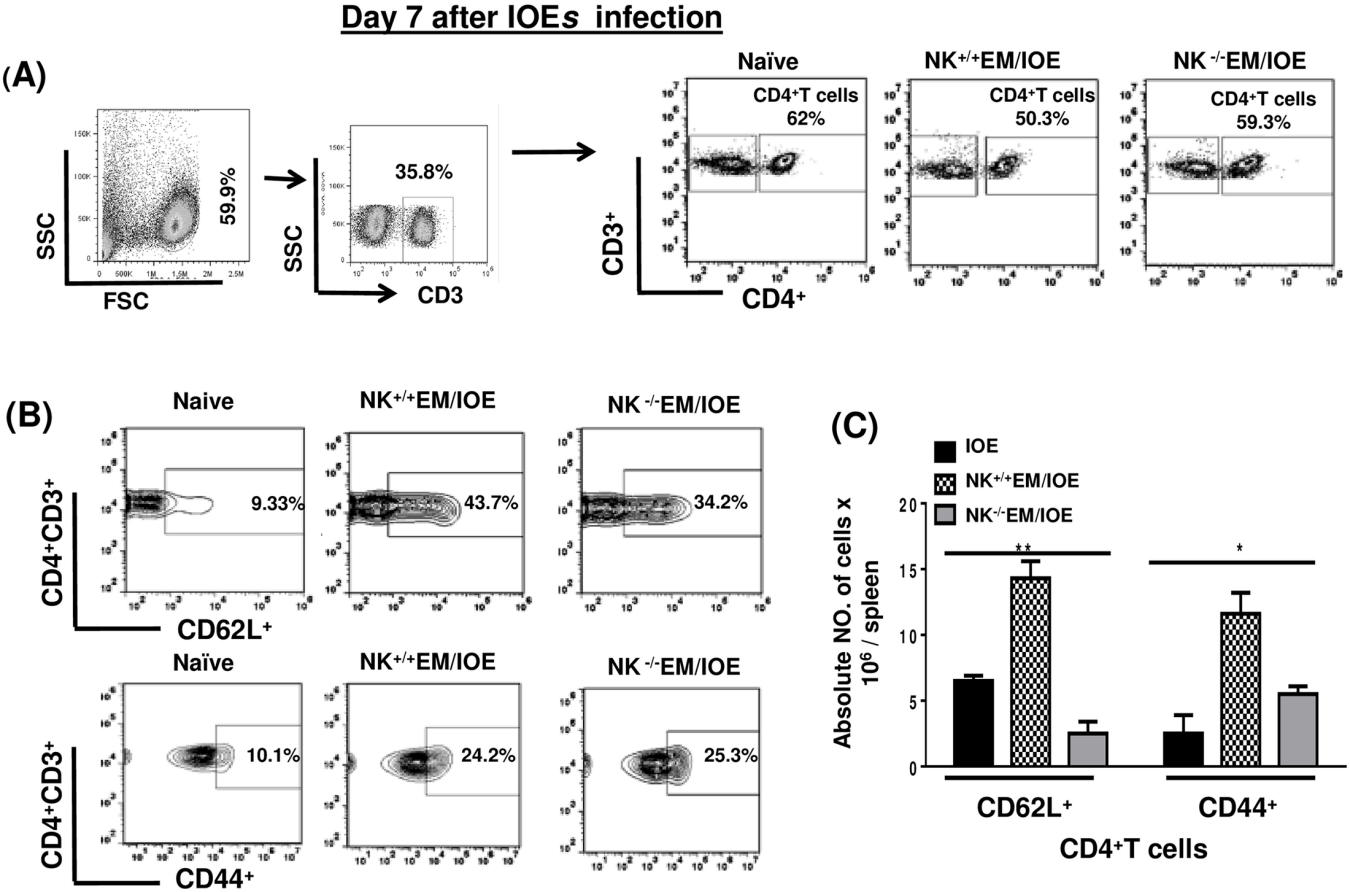
NK cell depletion in EM/IOE-infected mice impairs expansion of effector memory CD4^+^ T cells. Splenocytes harvested on day 7 after IOE infection from NK^-/-^EM/IOE and NK^+/+^EM/IOE-mice were stimulated *in vitro* with IOE antigens and the frequency of antigen-specific activated/effector memory T cells were determined by flow cytometry. Naïve cells were stimulated *in vitro* with IOE antigens. Gating on splenocytes and T cells was shown in **(A)**. The spleens of NK^-/-^EM/IOE-mice contained lower percentages **(B)** and absolute numbers **(C)** of CD62L^+^CD4^+^ T cells compared to that detected in the spleens of NK^+/+^EM/IOE-infected mice on day 7 post-IOE infection. Data are presented as the means ± SD of four mice per group from two independent experiments. ** indicates *P* < 0.01.

**Fig 6:**
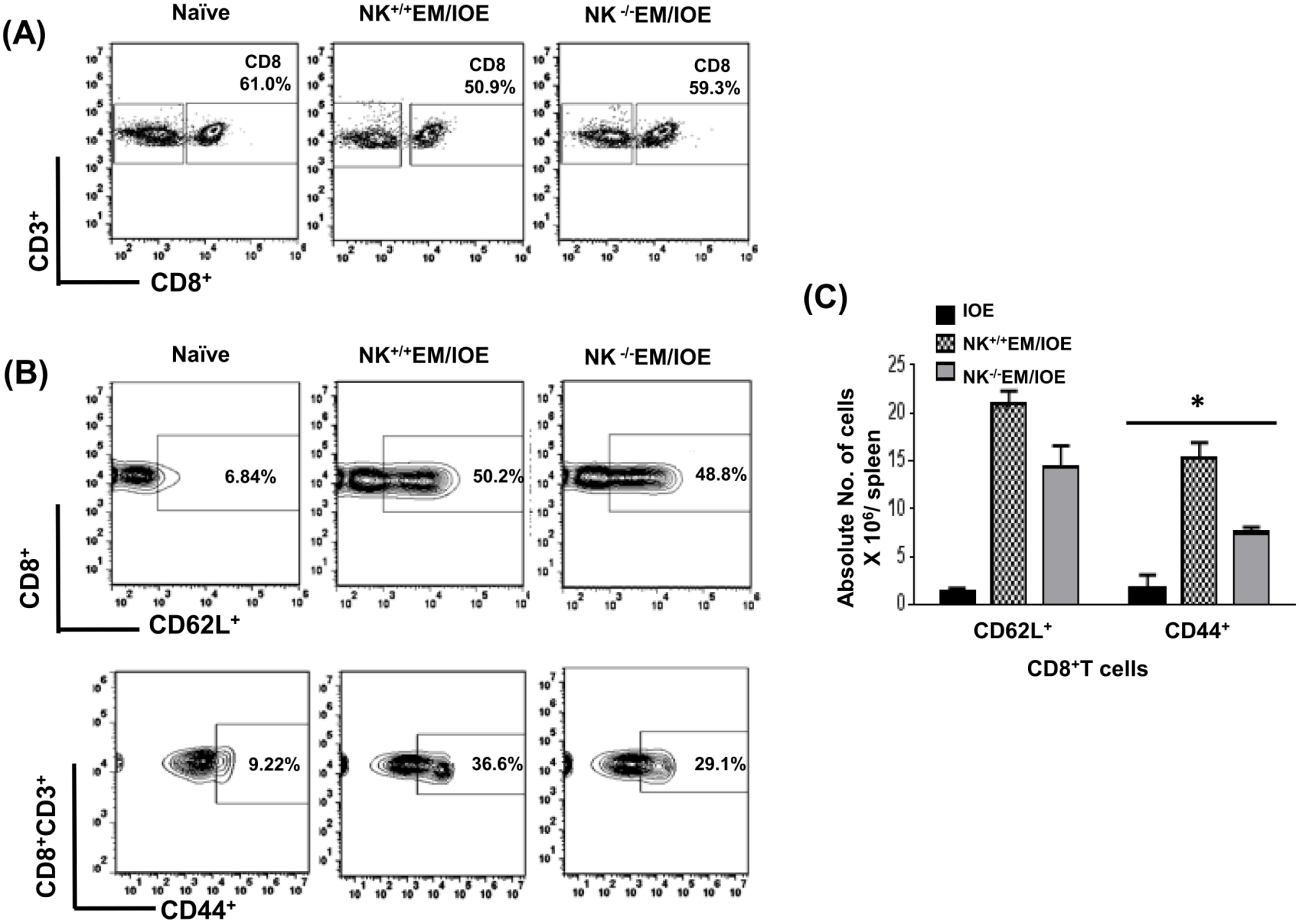
NK cell depletion in EM/IOE-infected mice impairs expansion of effector memory CD8^+^ T cells. Splenocytes harvested on day 7 after IOE infection from NK^-/-^EM/IOE and NK^+/+^EM/IOE-mice were stimulated *in vitro* with IOE antigens and the frequency of antigen-specific activated/effector memory T cells were determined by flow cytometry. Naïve cells were stimulated *in vitro* with IOE antigens. Gating on splenocytes and T cells was shown in **(A)**. The spleens of NK^-/-^EM/IOE-mice contained lower percentages **(B)** and absolute number **(C)** of CD44^+^ CD8^+^ T cells compared to that detected in the spleens of NK^+/+^EM/IOE-infected mice. No significant difference in the frequency of CD62L^+^ CD8^+^ T cells between NK^-/-^EM/IOE-mice and NK^+/+^EM/IOE-mice. Data are presented as the means ± SD of four mice per group from two independent experiments. ** indicates *P* < 0.01.

To determine the effect of NK cell depletion on the frequency of activated/effector memory CD4^+^ T cells following secondary IOE infection, we measured the expression of CD62L and CD44 on T cells in the spleen of NK^-/-^EM/IOE and NK^+/+^EM/IOE mice 7 DPI after IOE infection. Following *in vitro* stimulation with IOE antigens, we detected lower a percentage and absolute number of activated CD62L^+^ and effector/effector memory CD44^+^CD4^+^T cells in the spleen of NK^-/-^EM/IOE mice than that detected in the spleen of NK^+/+^EM/IOE mice Fig 5A–5C. NK cell depletion also significantly decreased the percentage and absolute number Fig 6A–6C of effector/effector memory CD44^+^CD8^+^ T cells, but not CD62L^+^CD8^+^ T cells, in NK^-/-^EM/IOE mice when compared to NK^+/+^EM/IOE mice. *In vitro* stimulation of naïve splenocytes with IOE or *E*. *muris* (data not shown) did not elicit significant activation of CD4^+^ T cells or naïve CD8^+^ T cells as compared to primed mice. Together, these data suggest that NK cells promote expansion of effector/effector memory CD4^+^ T cells and CD8^+^ T cells following primary and secondary *Ehrlichia* infection.

### Impaired protective immunity in NK cell-depleted, EM/IOE-infected mice is due to decreased IFN-γ, iNOS and NO

**Fig 7:**
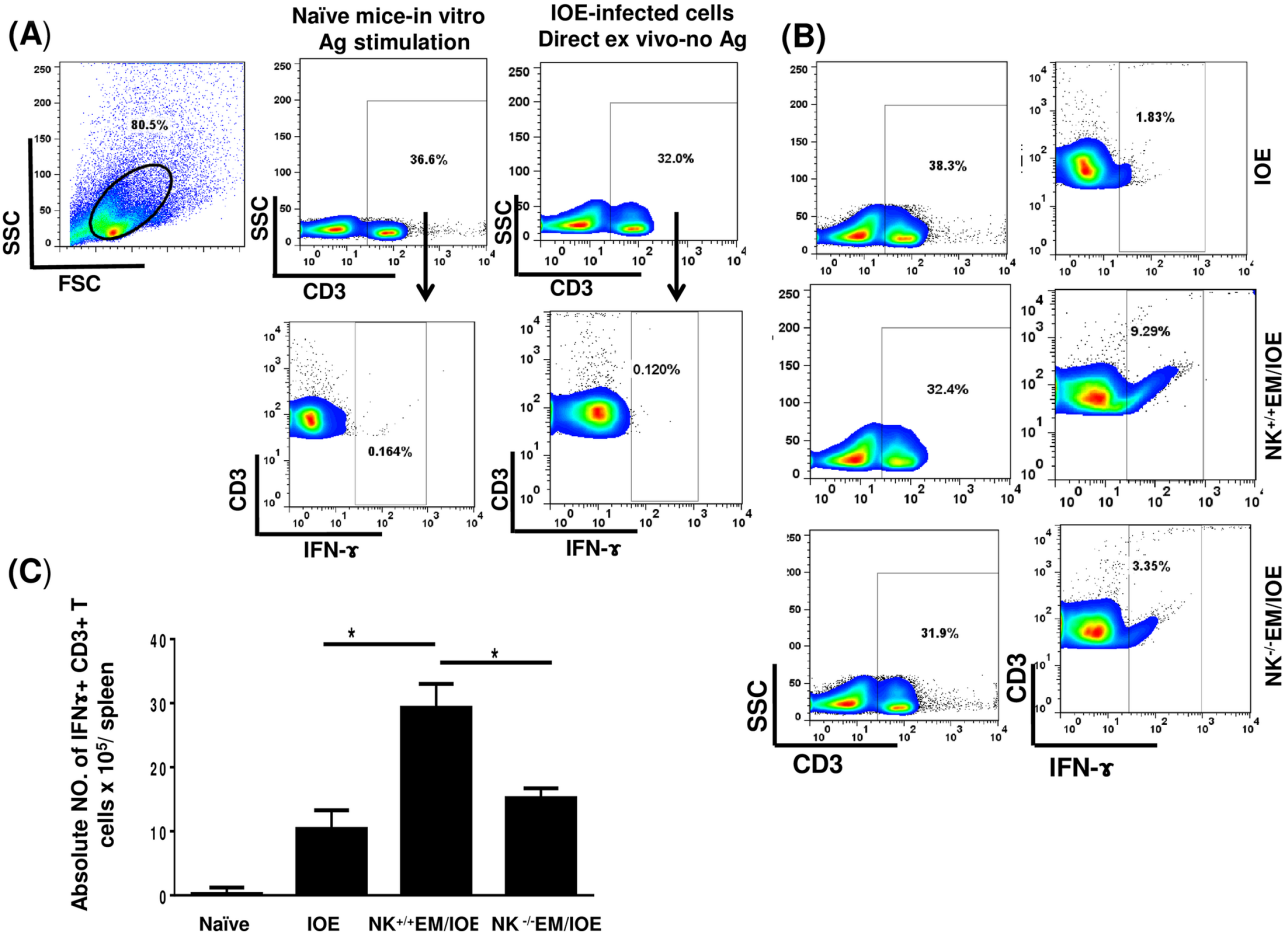
Decreased percentage of IFN-γ producing CD3^+^ T cells in NK^-/-^EM/IOE-mice. Splenocytes were harvested from the indicated mice groups on day 7 after IOE infection, and were stimulated *in vitro* with *E*. *muris* Ags or left unstimulated. Lymphocytes were gated based on the forward and side scatter, and then cells were gated on CD3. CD3^+^ T cells were further analyzed for intracellular IFN-γ staining as shown in the gating strategy **(A)**. Controls shown in dot plots includes naïve splenocytes stimulated *in vitro* with *E*. *muris* Ags and IFN-γ production by unstimulated splenocytes from IOE-infected mice. **(B)** Dot plots shows the percentage of gated CD3^+^ T cells and the percentage of IFN-γ^+^ CD3^+^ T cell subset in indicated mice groups following *in vitro* stimulation with *E*. *muris* Ags. (C) Data show the differences in the absolute number of IFN-γ^+^ CD3^+^ T cells in the spleen of indicated mice groups. Data shown are from three mice/groups with similar results in three independent experiments (n = 9 mice/group).

**Fig 8:**
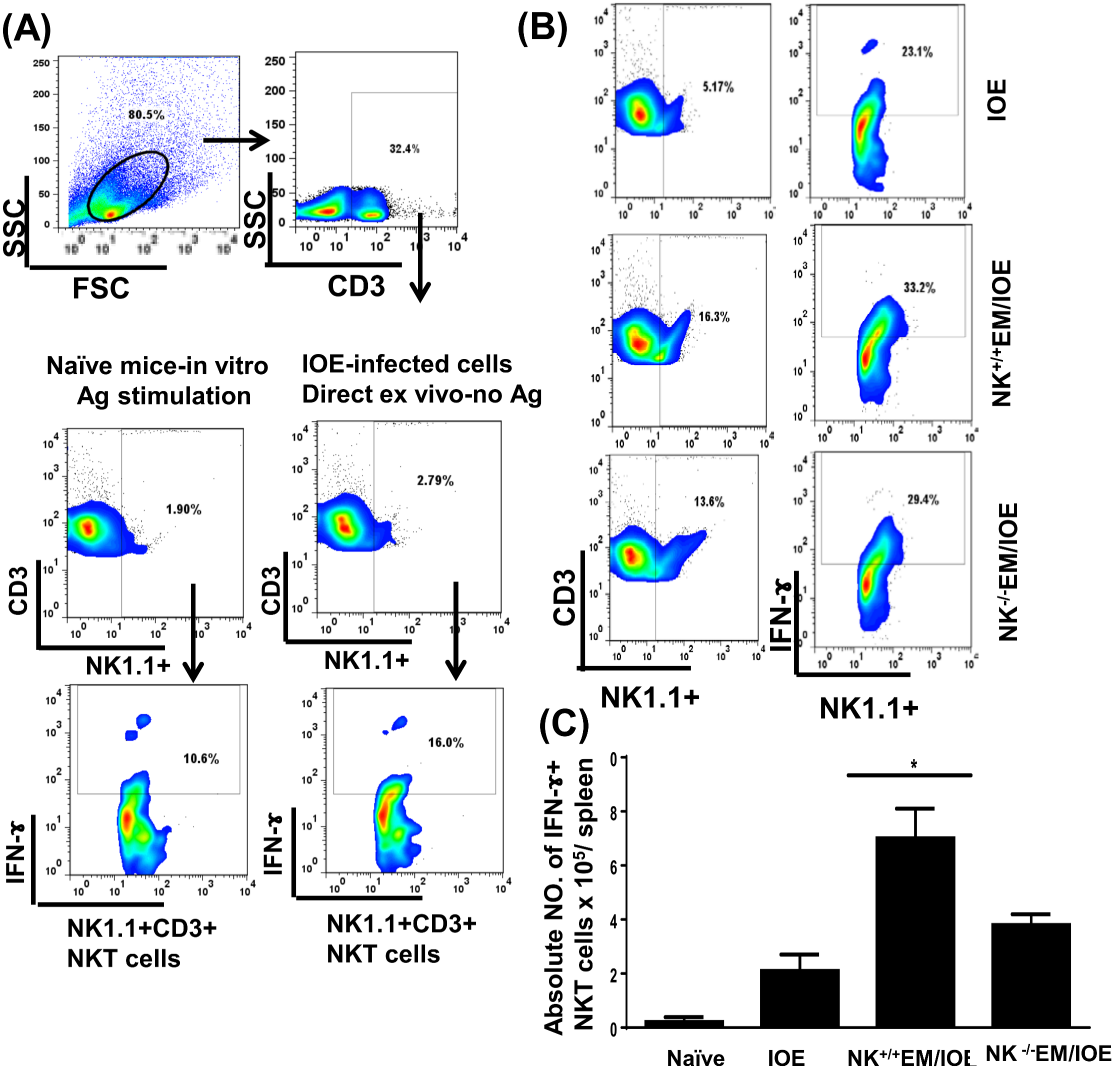
Decreased percentage of IFN-γ producing NKT cells in NK^-/-^EM/IOE-mice. Splenocytes were harvested from the indicated mice groups on day 7 after IOE infection, and were stimulated *in vitro* with *E*. *muris* Ags or left unstimulated. (A) dot plots show the gating strategy where lymphocytes were first gated based on the forward and side scatter, and then cells were gated on CD3. CD3^+^ T cells were further gated on NK1.1.^+^ cells (NKT cells). The NKT cells were further analyzed for intracellular IFN-γ staining. Controls shown in the dot plots includes naïve splenocytes stimulated *in vitro* with *E*. *muris* Ags and IFN-γ production by unstimulated splenocytes from IOE-infected mice. **(B)** Dot plots shows the percentage of gated CD3^+^ T cells and the percentage of IFN-γ^+^ producing NKT cells in indicated mice groups following *in vitro* stimulation with *E*. *muris* Ags. (C) Data show the differences in the absolute number of IFN-γ^+^ producing NKT cells in the spleens of indicated mice groups. Data shown are from three mice/groups with similar results in three independent experiments (n = 9 mice/group). * indicates *P* <0.05.

IFN-γ is critical for effective bacterial elimination in ehrlichiosis. T cells, particularly CD4^+^ Th1 cells and NKT cells are major cellular subsets that produce IFN-γ and thus mediate protective immunity against *Ehrlichia*. To determine the role of NK cells in host defense against *Ehrlichia* during recall response, we examined the number of antigen-specific, IFN-γ-producing T cells and NKT cells in all mice groups on day 7 after the second IOE infection. We stimulated splenocytes from naïve and infected mice with *E*. *muris* Ags, and the frequency of *E*. *muris*-specific, IFN-γ-producing cells was determined by flow cytometry. Impaired protective immunity in NK^-/-^EM/IOE mice was associated with decreases in the percentage Fig 7A and 7B and absolute number Fig 7C of *E*. *muris*-specific IFN-γ-producing CD3^+^ T cells when compared with those found in NK^+/+^EM/IOE control mice. Similarly, the percentage Fig 8A and 8B and absolute number Fig 8C of IFN-γ-producing NKT cells was decreased in NK^-/-^EM/IOE mice compared to NK^+/+^EM/IOE mice. Polyclonal stimulation of splenocytes from all mice (naïve and infected) groups with PMA/ionomycin resulted in a similar increase in the number of IFN-γ-producing T cells (data not shown), suggesting that an impaired memory CD4^+^ Th1 response in NK^-/-^ EM/IOE mice encompasses antigen-specific T cells. We did not find a significant difference in the frequency of antigen-specific, IL-4-producing cells or IL-10-producing T cells between NK^+/+^EM/IOE and NK^-/-^EM/IOE-infected mice (data not shown), suggesting that impaired bacterial elimination is not due to altered IL-4: IFN-γ or IL-10:IFN-γ responses, as suggested by other studies [18,21,26,27].

**Fig 9:**
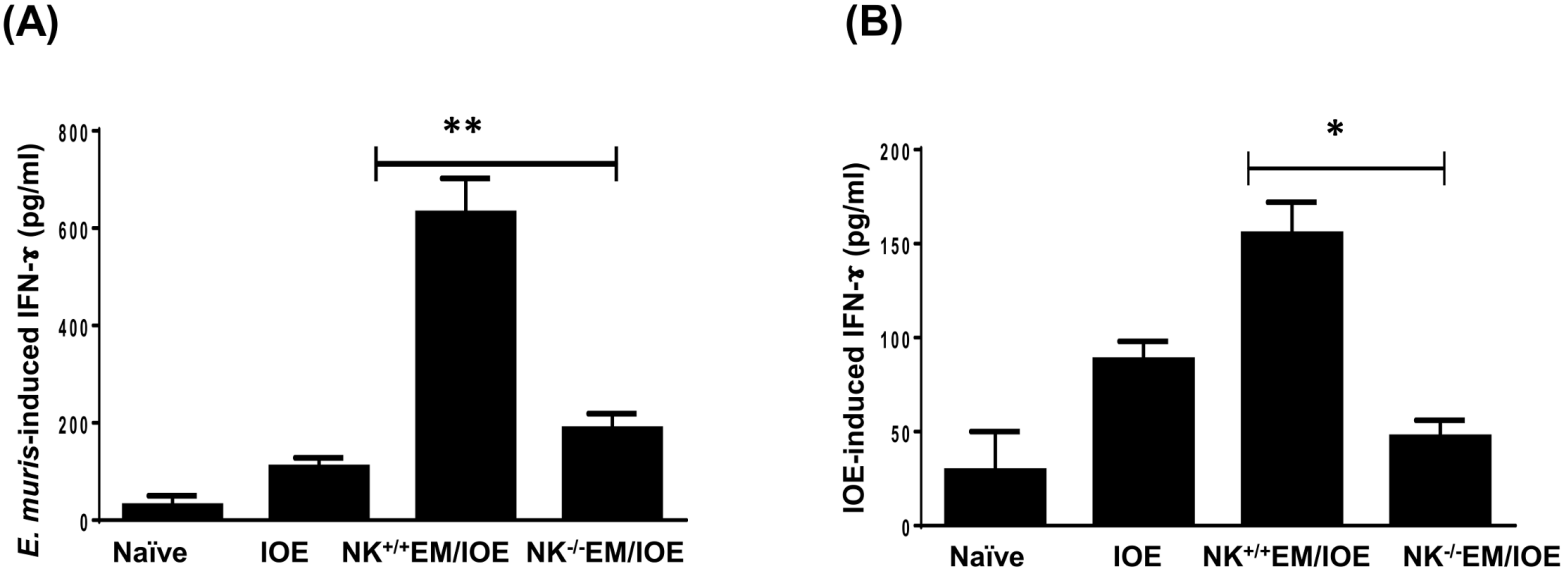
Depletion of NK cells in NK^-/-^EM/IOE mice decreased production of IFN-γ. The levels of IFN-γ in bulk culture of splenocytes from the indicated mice groups, harvested on day 7 after IOE infection, and stimulated *in vitro* with either *E*. *muris* (A) or IOE (B) Ags, were measured by ELISA. The antigen-specific IFN-γ response was calculated by subtraction of the IFN-γ concentration produced by unstimulated cells from the Ags-stimulated cells. The data show a significantly lower production of *E*. *muris*- **(A)** and IOE- **(B)** specific IFN-γ by splenocytes from NK^-/-^EM/IOE mice compared with similarly-stimulated cells from NK^+/+^EM/IOE mice. The levels of IFN-γ in NK^-/-^EM/IOE-mice were similar to those detected in naïve mice infected with IOE. * and ** indicate *P* < 0.05 and *P* < 0.01, respectively. Data are representative of two independent experiments with four mice per group.

To determine the difference in total IFN-y production in the spleen of NK cell-depleted and undepleted primed mice, we stimulated splenocytes from all mice groups with *E*. *muris* and IOE Ags, and measured IFN-γ levels in bulk culture supernatants by ELISA. Although *in vitro* stimulation of immune splenocytes with either *E*. *muris* or IOE Ags induced higher IFN-γ production as compared to that produced by naïve splenocytes, the levels of IFN-γ were lower when immune splenocytes were stimulated with IOE Ags. This is not surprising since our previous studies show that IOE is a poor inducer of IFN-γ response. Nevertheless, depletion of NK cells decreased the level of *E*. *muris*- Fig 9A or IOE- Fig 9B induced IFN-γ produced in the spleen of NK^+/+^EM/IOE-infected mice compared to that produced by splenocytes from NK^-/-^EM/IOE mice. Ag-specific IFN-γ production by the spleen of NK^-/-^EM/IOE mice was similar to that produced in the splenocytes cultured from unprimed, IOE-infected mice, suggesting that the absence of NK cells abrogated the protective memory response against *Ehrlichia*.

**Fig 10:**
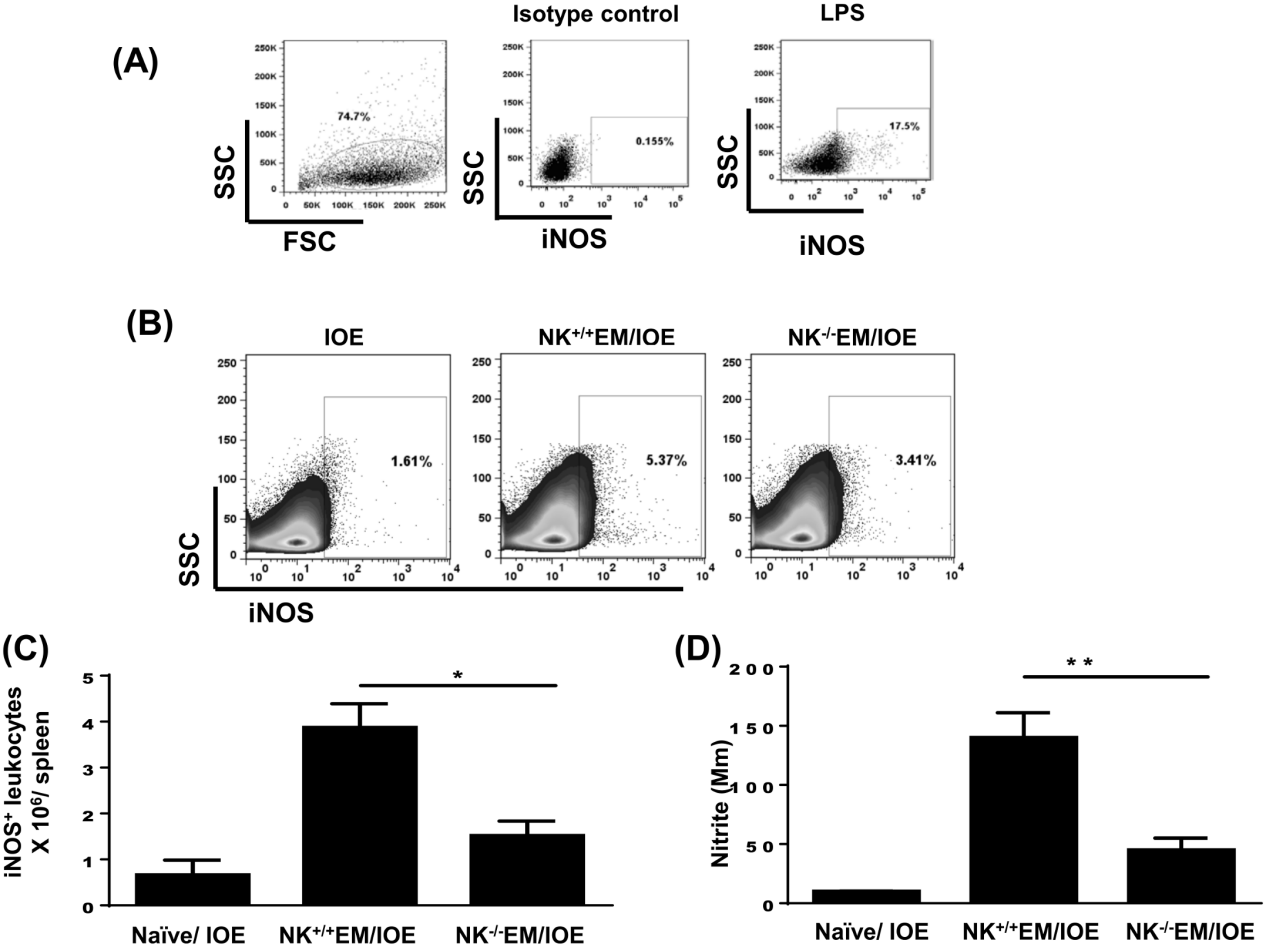
Depletion of NK cells in NK^-/-^EM/IOE mice decreased production of iNOS and NO. Spleen cells were harvested from the indicated mice groups on day 7 after IOE infection, and were stimulated *in vitro* with *E*. *muris* Ags or left unstimulated. **(A)** Dot plots shows negative control (cells stained with isotype control Ab) and positive control (splenocytes from IOE-infected mice stimulated *in vitro* with LPS). Cells were gated on leukocytes based on forward and side scatter. (**B and C**) Data show lower percentages and absolute number, respectively, of iNOS-producing leukocytes compared with NK^+/+^EM/IOE mice. **(D)** The level of NO produced by *E*. *muris*-stimulated cells was measured as described in Materials and Methods. Data show a significantly lower production of NO by splenocytes from NK^-/-^EM/IOE mice compared with NK^+/+^EM/IOE mice. The levels of NO in NK^-/-^EM/IOE-mice were similar to those detected in naïve mice infected with IOE. * and ** indicate *P* < 0.05 and *P* < 0.01, respectively. Data are representative of two independent experiments with four mice per group.

IFN-γ is known to mediate activation of the bactericidal functions of macrophages, such as the induction of inducible nitric oxide synthase (iNOS) and production of nitric oxide (NO). The latter is a key antimicrobial effector molecule. Thus, we examined whether the difference in IFN-γ response between NK depleted and NK sufficient mice influenced the production of iNOS and NO. We measured the number of CD45^+^ leukocytes producing iNOS and the quantity of nitrites in the culture supernatants by flow cytometry and Griess reaction, respectively. Negligible iNOS production was detected in isotype control mAb- stained splenocytes from IOE-infected mice (negative control) Fig 10A, while a significantly high percentage of iNOS-producing cells were detected when IOE-infected splenocytes were further stimulated *in vitro* with lipopolysaccharide (LPS) (positive control) Fig 10A. Our data showed that the spleens of NK^+/+^EM/IOE-infected mice contain a higher percentage Fig 10B and absolute number Fig 10C of iNOS-producing leukocytes when compared to IOE-infected mice and NK^-/-^EM/IOE. Consistent with single cell analysis, there was a higher production of nitrite in spleen bulk culture in NK^+/+^EM/IOE-infected mice compared to other mice groups Fig 10D. Together, these data suggest that NK cells promote bacterial elimination and the protective memory response against *Ehrlichia* by enhancing the induction and/or expansion of effector memory CD4^+^ Th1 cells, IFN-γ production, and the activation of the bactericidal function of infected phagocytic cells.

### NK cells are required for the production of *Ehrlichia*-specific antibodies

*Ehrlichia*-specific antibodies, mainly IgG2a, are critical for the elimination of intracellular *Ehrlichiae* by enhancing opsonization of extracellular *Ehrlichiae* and subsequent intracellular killing within phagocytes. Thus, we examined the effect of NK depletion on the production of Ag-specific antibodies in EM/IOE-infected mice following primary infection with *E*. *muris* or after re-challenge with IOE. The levels of antibodies in different mice groups were measured as the reciprocal of the dilution of serum samples. Similar to our previous findings [6,23,25], primary infection with *E*. *muris* induced a high Ag-specific IgG titer 21 DPI (titer is 512). NK^+/+^ EM/IOE-infected control mice had substantially high titers of *Ehrlichia*-specific IgG antibodies (titer is 1024) on day 7 post IOE challenge. In contrast, NK^-/-^ EM/IOE mice had a lower level of *Ehrlichia*-specific IgG antibodies (titer is 128) on day 7 post IOE re-challenge.

**Fig 11:**
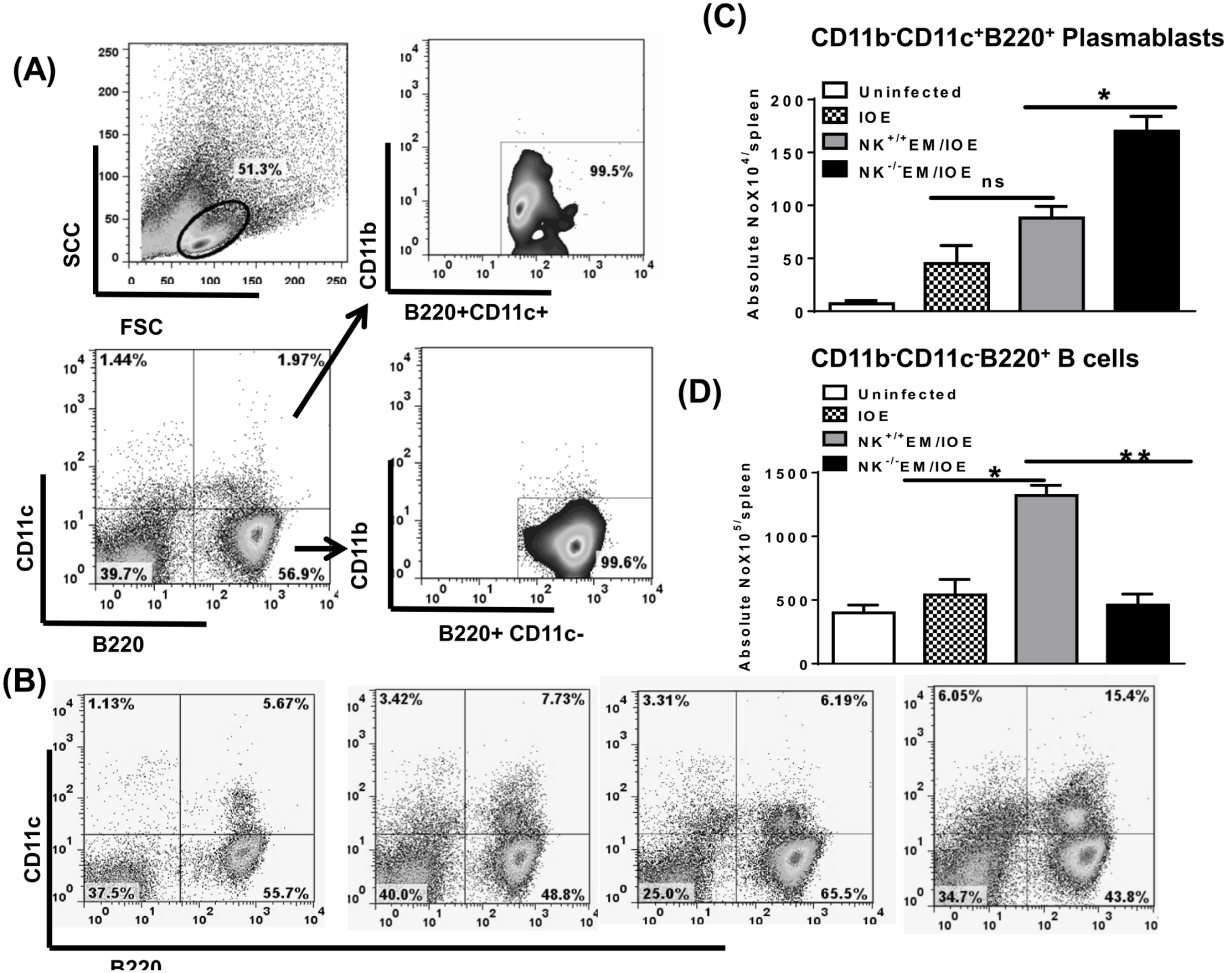
NK cell depletion negatively affects antibody production and B cell expansion during memory response to *Ehrlichia*. Splenocytes were harvested from uninfected mice, IOE-infected mice, NK^-/-^EM/IOE-mice, and NK^+/+^EM/IOE mice on day 7 after IOE infection. Splenocytes from all groups were stimulated *in vitro* with IOE Ags. (A) Shows gating strategy on negative controls (naïve splenocytes that are not stimulated *in vitro* with Ags). Splenic mononuclear cells were gated based on forward and side scatter and then further analyzed for the expression of CD11c and B220. B220^+^CD11c^+^ and B220^+^ CD11c^+^cells were further analyzed for the expression of CD11b to define two cellular subsets; Plasmablasts (B220^+^CD11c^+^CD11b^-^) and B cells (B220^+^CD11c^+^ CD11b^-^). The percentages **(B**) and absolute numbers **(C)** of CD11b^-^CD11c^+^B220^+^ plasmablasts in NK^-/-^EM/IOE mice were significantly higher than the other groups of mice. The percentages **(B**) and absolute numbers **(D)** of CD11b^-^CD11c^-^B220^+^ B cells in NK^-/-^EM/IOE mice were significantly lower than the numbers of B cells in NK^+/+^EM/IOE but similar to those detected in unprimed mice infected with IOE. * and ** indicate *P* < 0.05 and *P* < 0.01, respectively. Data are presented as the means and SD of three mice per group and are representative of two independent experiments.

Studies conducted by Winslow et al. have shown that chronic *E*. *muris* infection elicits a protective IgM response derived from extrafollicular CD11b^-^CD11c^+^B220^+^ plasmablasts. We found that primary or secondary IOE infection in naïve or *E*. *muris*–primed mice induced similar antigen-dependent expansion of CD11b^-^CD11c^+^B220^+^ plasmablasts in the spleen on day 7 after IOE infection when compared to *in vitro* Ag-stimulated splenocytes from naïve mice Fig 11A–11C. Interestingly, depletion of NK cells in NK^-/-^ EM/IOE-infected mice significantly increased the frequency of these plasmablasts compared with NK^+/+^ EM/IOE-infected mice Fig 11A–11C. On the other hand, decreased antibody productions in NK^-/-^ EM/IOE mice were associated with decreased percentage Fig 11A and 11B as well as absolute number Fig 11D of antigen-specific CD11b^-^CD11c^-^B220^+^ mature B cells. These data further suggest that chronic *E*. *muris* infection elicits NK cells that regulate antigen-specific T and B cell responses against *Ehrlichia*.

### Depletion of NK cells in *E*. *muris*-primed mice enhanced liver injury following lethal IOE infection

**Fig 12:**
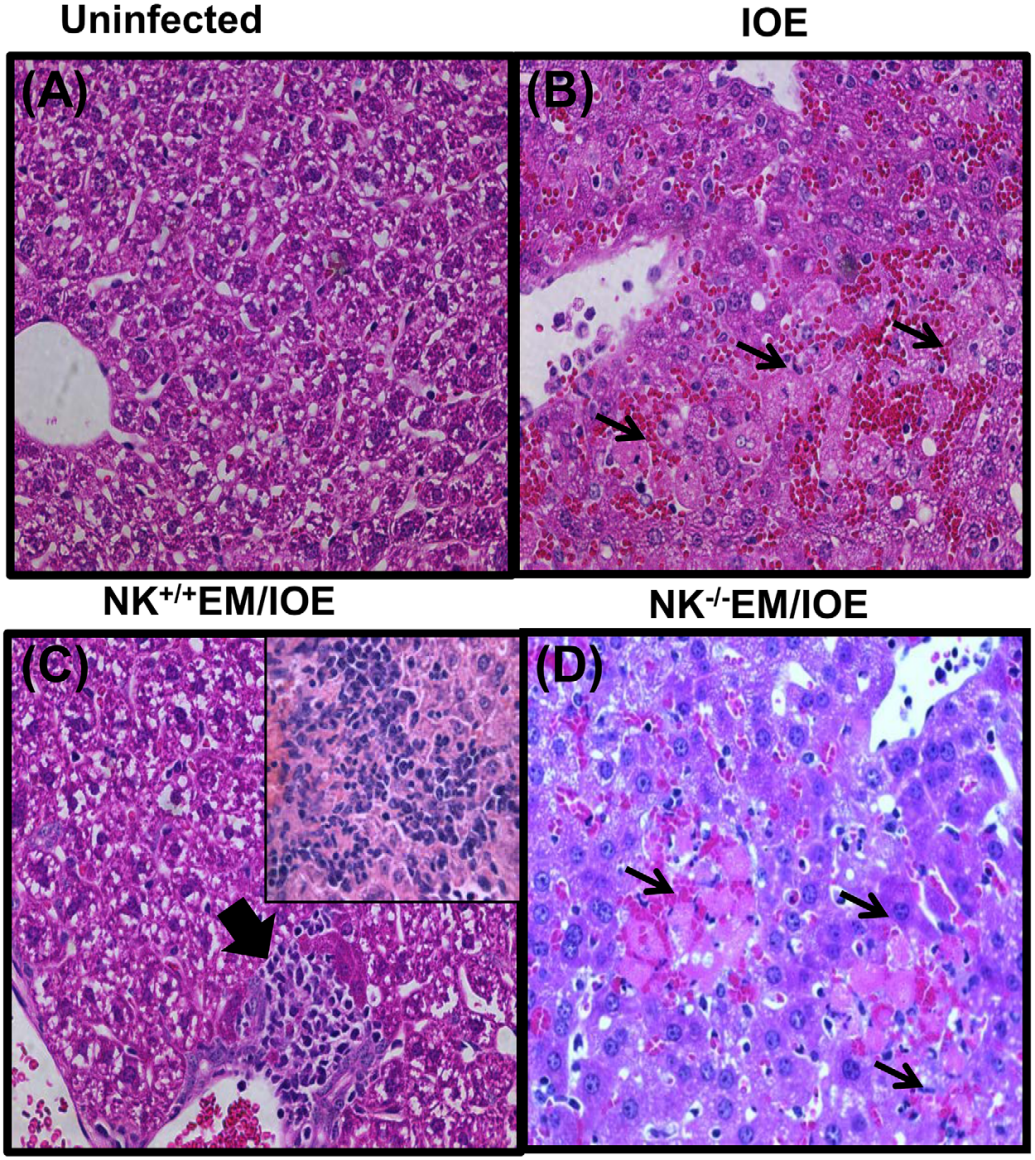
NK cells confer protection against IOE-mediated immunopathology during recall response. Compared with naïve mice **(A),** and IOE-infected/unprimed mice **(B),** the livers of NK^+/+^EM/IOE mice **(C)** have minimal apoptotic and necrotic cells but contain lymphohistiocytic cellular infiltration (arrowhead and inset) and minimal apoptosis. In contrast, the livers of NK^-/-^EM/IOE-infected mice **(D)** has a greater number of apoptotic cells and foci of liver necrosis (arrows), as detected by H&E staining after challenge with IOE. (Original magnification, X40). Data are representative of sections from one mouse in each group with similar results obtained in three independent experiments with three mice per group.

We previously showed that primary and secondary fatal ehrlichiosis is due to excessive inflammation, which is mediated in part by TNF-α and leads to extensive liver injury [6,10,25,28]. Thus, we examined the liver pathology of undepleted and NK cell-depleted EM/IOE mice 7 DPI by H&E staining. Compared to uninfected controls Fig 12A and unprimed/IOE-infected mice Fig 12B, NK^+/+^EM/IOE-infected mice developed prominent lymphohistiocytic infiltrates in the liver, which were associated with minimal necrosis and apoptosis Fig 12C. However, NK^-/-^EM/IOE-infected mice exhibited focal areas of confluent necrosis, extensive apoptosis of Kupffer cells and hepatocytes, and microvesicular steatosis and congestion 7–10 days post IOE infection Fig 12D, which was similar to that detected in unprimed, IOE-infected mice Fig 12B. These data suggest that NK cells prevent the development of pathology and liver injury during the recall response to *Ehrlichia* infection.

### *E*. *muris* induces an optimal cytokine environment that promotes the induction and maintenance of memory-like NK cells

**Fig 13:**
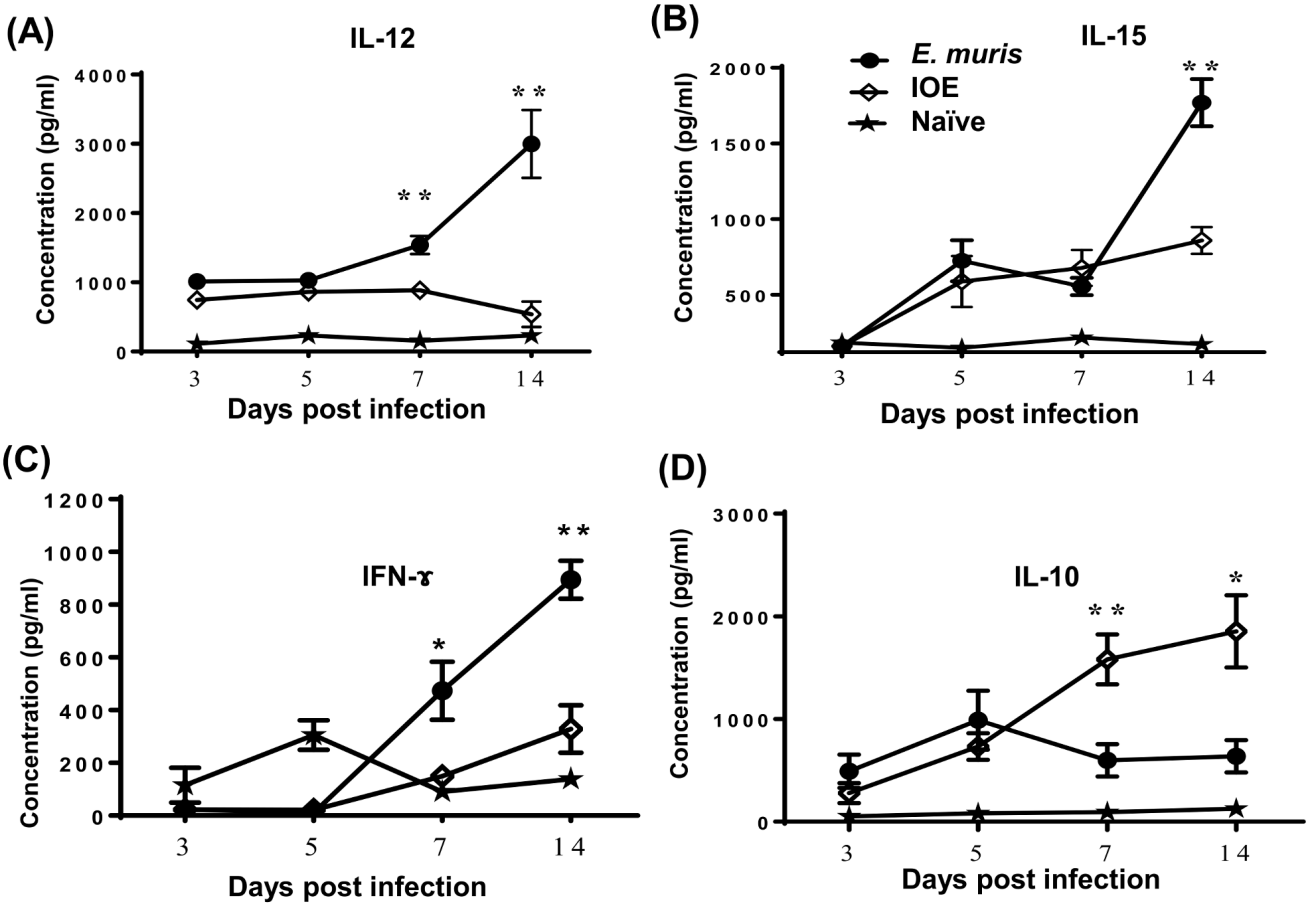
*E*. *muris* induces an optimal cytokine environment that promotes induction and maintenance of memory-like NK cells. Cultured supernatants of liver mononuclear cells (LMNCs) harvested 3, 5, 7 and 14 DPI from *E*. *muris*-primed mice and stimulated *in vitro* with *E*. *muris* Ag contained significantly higher levels of IL-12 **(A)**, IL-15 **(B)** and IFN-γ **(C)** compared to LMNCs from naïve mice and IOE-primed mice. On the other hand, LMNC culture supernatant from IOE-primed mice contained higher levels of IL-10 as compared to naïve mice and *E*. *muris*-primed mice **(D).** *, ** indicate *P* < 0.05 and *P* < 0.01, respectively. Data are presented as the means ± SD of 3 mice/ group and are representative of two independent experiments.

Recent studies have suggested that NK cells previously activated by cytokines or by ligation of their activating NK receptors (including FcR) display memory-like phenotypes, which cause them to respond more robustly to reactivation [29]. Because primary infection with a low dose of IOE does not confer a protective memory response, we hypothesized that *E*. *muris* infection may differentially activate innate immune cells and induce a different cytokine environment that promotes the differentiation of NK cells into a memory phenotype. Thus, we examined the levels of cytokines that are known to promote the survival, proliferation, IFN-γ production and cytotoxicity of NK cells, namely IL-12, IL-15, and IL-18, in the liver of infected mice. We also measured the level of IL-10 in these mice, which is known to impair protective primary and memory immune responses against *Ehrlichia* [26,30]. LMNCs from *E*. *muris*-infected mice were harvested 3, 5, 7 and 14 DPI and cells were stimulated with *E*. *muris* Ags. Compared with IOE-infected mice, LMNCs from *E*. *muris*-infected mice secreted higher levels of IL-12 and IFN-γ at 7 and 14 DPI and a higher level of IL-15 at 14 DPI. Fig 13A–13C. In contrast, we detected higher production of IL-10 by LMNCs from IOE-infected mice at 7 and 14 DPI as compared with *E*. *muris*-infected mice Fig 13D.

### NK cells in *E*. *muris*-primed mice acquire adaptive features and a memory phenotype

**Fig 14:**
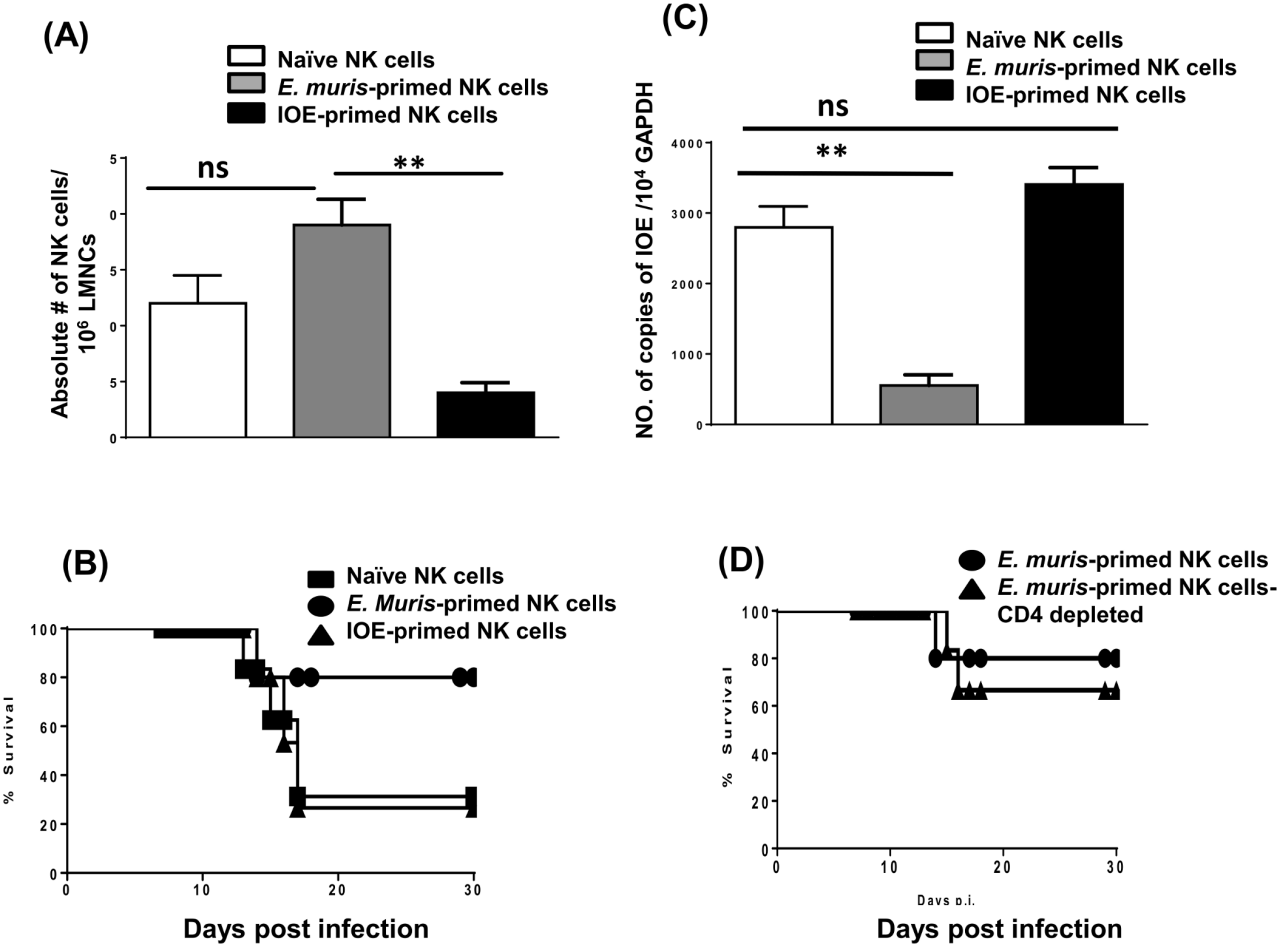
NK cells in *E*. *muris*-primed mice acquire adaptive features and a memory phenotype. CD45^+^NK1.1^+^CD3^-^ NK cells were purified from the spleen and liver collected on day 21 from *E*. *muris*-primed mice, and ~5–8 x 10^5^ splenic and hepatic NK cells (1:1 ratio) were transferred into naïve *Rag2*^−/−^*Il2rg*^−/−^ mice. Control mice were naïve *Rag2*^−/−^*Il2rg*^−/−^ hosts receiving naïve NK cells. The numbers of memory-like NK cells, as measured by flow cytometry, in recipient *Rag2*^−/−^*Il2rg*^−/−^ mice that received naïve, *E*. *muris*-primed or IOE-primed NK cells **(A)**. *Rag2*^−/−^*Il2rg*^−/−^ hosts receiving *E*. *muris-* primed NK cells survived longer than mice receiving naïve or IOE-primed NK cells following *E*. *muris* infection **(B)**. Bacterial burden in the livers of recipient *Rag2*^−/−^*Il2rg*^−/−^ mice at day 7 following *E*. *muris* infection **(C)**. Depletion of contaminating donor CD4^+^T cells in recipient *Rag2*^−/−^*Il2rg*^−/−^ mice did not influence mice survival following *E*. *muris* infection **(D)**. ** indicate *P* < 0.01. Data are presented as the means ± SD of 3 mice/ group and are representative of two independent experiments. Data are presented as means ± SD from three mice per group. Data shown are representative of three independent experiments.

We then examined whether *E*. *muris*-primed NK cells acquire features of memory cells, namely prolonged survival, in an antigen-free hosts and providing recall response. To this end, we adoptively transferred purified NK cells collected on day 21 from the spleens and livers of *E*. *muris*-primed and IOE-primed mice (~5–8 x 10^5^ cells; 1:1 ratio) into recipient *Rag2*^−/−^*Il2rg*^−/−^ mice (which lack T, B and NK cells). Control mice were naïve *Rag2*^−/−^*Il2rg*^−/−^ hosts receiving naïve NK cells. NK cells from *E*. *muris*-primed mice were detected in the liver of *Rag2*^−/−^*Il2rg*^−/−^ mice on day 7 after transfer, similar to the observations made in naïve NK cells Fig 14A. Donor NK cells from IOE-primed mice were detected in the liver of *Rag2*^−/−^*Il2rg*^−/−^ mice on day 7 after transfer Fig 14A. However, the number of these cells was significantly lower compared to mice receiving naïve NK cells and *E*. *muris*-primed NK cells, suggesting that these primed cells may not be able to survive in lymphopenic host. Taken all together, these data suggest that NK cells from *E*. *muris*-primed mice may acquire memory-like phenotype and thus able to survive in lymphopenic hosts and in the absence of T and B cells, and in the absence of persistent *E*. *muris* infection

To examine whether these NK cells provide a protective memory response against *Ehrlichia* in lymphopenic hosts, we challenged recipient mice 24 h after transfer with a high dose of *E*. *muris* (10^4^ bacteria/mouse). The infection of *Rag2*^−/−^*Il2rg*^−/−^ mice receiving *E*. *muris*-primed NK cells with *E*. *muris* resulted in survival rate of a ~75% of the recipient mice transferred with *E*. *muris*-primed NK cells as compared to ~30% survival rate of *Rag2*^−/−^*Il2rg*^−/−^ mice receiving IOE-primed NK cells Fig 14B. Protection of mice transferred with *E*. *muris*-primed NK cells infection was associated with a lower bacterial burden in the liver of these mice as compared with mice receiving naïve NK cells Fig 14C. Similar to control mice receiving naïve NK cells, *Rag2*^−/−^*Il2rg*^−/−^ mice receiving IOE-primed NK cells have a high bacterial burden, suggesting that primed donor NK cells from these mice are either dysfunctional or ineffective in providing protective response against heterologous *E*. *muris* challenge.

Since donor NK cells were not 100% pure, we examined whether protection of *Rag2*^−/−^*Il2rg*^−/−^ mice was mediated, in part, by the contaminating CD4^+^ lymphocytes transferred from donor *E*. *muris*-primed WT mice. To that end, we transferred purified *E*. *muris*-primed NK cells, and then treated recipient mice with anti-CD4 mAb at -1, 0, and 3 DPI. As shown in Fig 14D, depletion of contaminating CD4^+^ lymphocytes did not influence the survival of recipient mice following *E*. *muris* infection, suggesting that protective memory response in the *Rag2*^−/−^*Il2rg*^−/−^ recipient mice is indeed mediated by memory-like NK cells. Together, these data suggest that *E*. *muris* infection induces memory-like NK cells and that the survival and maintenance of these NK cells does not require persistent infection.

## Discussion

Natural killer (NK) cells are innate lymphoid cells that play a role in host defense against several bacterial and viral pathogens via the production of IFN-γ and cytotoxic killing of target cells [31–34]. NK cells express germline-encoded activating and inhibitory receptors and are able to respond to a diverse range of signals during their interaction with their target cells. In this study, we provide the first demonstration that NK cells generated upon primary infection with intracellular *Ehrlichia* acquire adaptive features that include a robust recall response, antigen specificity and survival. Our study demonstrates that the ability of primary infection with *E*. *muris*, but not IOE, to provide a protective recall response is partially due to the expansion of memory-like NK cells in the liver and spleen of *E*. *muris*-primed mice and their persistence, even after clearance of the primary infection. The activation status of NK cells depends on the expression of activating and inhibitory receptors. Hepatic NK cells in *E*. *muris*-primed mice analyzed three weeks after infection expressed the activating receptor NKG2D, suggesting that memory-like NK cells were activated.

Mechanistically, we found that *E*. *muris*-primed, memory-like NK cells were critical for the expansion and survival of effector memory CD4^+^ T cells during the recall response to *Ehrlichia*. Memory CD4^+^ T cells are the major cell subset that mediates protective cellular and humoral memory responses against several intracellular pathogens, including *Ehrlichia*. Other studies have suggested that cognate and non-cognate interactions of NK cells with CD4^+^ T cells influence T cell activation, differentiation and adaptive immunity via multiple mechanisms [35–37]. Our previous studies demonstrated that primary *E*. *muris* infection induces the expansion and maintenance of *E*. *muris* specific central memory CD4^+^Th1 cells at 3–4 weeks after infection; the time points at which NK cells were depleted in the current study. We have also previously shown that central memory CD4^+^Th1 cells generated in *E*. *muris*-infected mice expand and differentiate into effector memory CD4^+^Th1 cells following a second IOE infection. Thus, it is possible that activated NK cells in *E*. *muris-* primed mice promote the induction/ maintenance of central memory CD4^+^Th1 cells, and/or expansion of effector memory CD4^+^Th1 cells following second IOE infection.

Although the mechanism through which NK cells promote the induction and/or maintenance of memory CD4^+^ T cells *in vivo* is not clearly known, it is possible that the interaction of NK cells and antigen-presenting cells, such as dendritic cells (DCs), enhances the T cell-costimulatory function of DCs. Alternatively, NK cells could produce soluble factors, such as IL-2 and IFN-γ, that enhance the proliferation and expansion of CD4^+^ Th1 cells. In support of the latter conclusion, we detected a higher level of NO produced by leukocytes in the spleen of NK^+/+^EM/IOE-infected mice as compared with NK^-/-^EM/IOE-infected mice. NO is an antimicrobial molecule produced by activated macrophages upon stimulation with IFN-γ and/or TNF-α. Thus, decreased IFN-γ production in NK^-/-^EM/IOE mice could result in decreased proliferation and differentiation of Th1 cells as well as ineffective activation of macrophages. Indeed, this was the case, as demonstrated by the finding that NK^-/-^EM/IOE mice had a lower frequency of *Ehrlichia*-specific, IFN-γ-producing CD4^+^ Th1 cells than the NK^+/+^EM/IOE group. The lack of IFN-γ and iNOS is consistent with the impaired bacterial clearance observed in NK^-/-^EM/IOE mice compared with the NK^+/+^EM/IOE controls. Notably, we did not detect significant differences in the IL-10 levels among the groups (data not shown), which further confirms that the higher bacterial burden in NK^-/-^EM/IOE mice is not due to increased IL-10 production, as suggested by other studies [7,18,21,27,38–40]. Together, these data suggest that NK cells are required for the induction of a strong *in vivo* memory CD4^+^ Th1 response against *Ehrlichia*.

Studies by our group and other investigators have demonstrated critical synergistic roles of antibodies and CD4^+^ T cells in mediating long-term protection against fatal *Ehrlichia* infection. NK cells express abundant activating FcγRIII (CD16) receptors. The Fc-mediated binding of antibodies to FCγRIIIa on NK cells mediates ADCC, which lyses target cells that express the antigens recognized by these antibodies [41–44]. Our results show that the lack of NK cells in EM/IOE mice decreased the production of *Ehrlichia*-specific IgG antibodies following reinfection, suggesting that NK cells contribute to the humoral response to *Ehrlichia*. Notably, decreased Ag-specific antibodies in NK^-/-^EM/IOE mice correlated with decreased numbers of mature B cells. The exact mechanism that accounts for the decreased B cell expansion and production of Ag-specific IgG antibodies has not yet been examined. However, it is possible that the decreased antibody response is secondary to a decreased CD4^+^ T cell response in NK^-/-^EM/IOE mice because CD4^+^ T cells are the major helper cells that induce B cell responses and isotype switching. Alternatively, *E*. *muris*-primed NK cells can directly provide help to B cells, as suggested by other studies. Although we have not examined the contribution of plasmablasts to antibody production and/or host resistance to primary or secondary ehrlichiosis following IOE infection, our data suggest that these cells may play a role in host susceptibility to fatal ehrlichiosis. This conclusion is merely based on the observed association between the higher expansion of splenic plasmablasts NK^-/-^EM/IOE mice and development of fatal disease. However, further mechanistic experiments are warranted to support this conclusion.

We and other researchers have shown that primary fatal ehrlichiosis is due to immunopathology [8,21,28,39,45]. In this study, we show that memory-like NK cells prevent extensive tissue injury during the recall response to *Ehrlichia*, as evidenced by the presence of focal areas of confluent necrosis and apoptosis of Kupffer cells and hepatocytes in NK^-/-^EM/IOE mice compared with NK^+/+^EM/IOE mice. Our previous studies suggested that Foxp3^+^ Treg cells and TGF-β play roles in the prevention of tissue injury during secondary ehrlichiosis [25]. Studies have shown that NK cells promote the development of Tregs via the production of IL-2 as well as a cognate interaction with antigen-presenting cells (APCs). However, it remains unclear as to whether NK cells regulate the generation of Tregs during the recall response to *Ehrlichia*.

Moreover, we investigated the potential mechanisms that could account for the survival and expansion of memory-like NK cells with protective functions during *E*. *muris* infection. Recent studies have shown that activation by cytokines alone leads to the generation of NK cells with memory-like properties [29,46]. Thus, we measured the levels of IL-12, IL-15, and IL-18, which are cytokines known to affect the survival, proliferation, IFN-γ production and cytotoxicity of NK cells. The *in vitro* stimulation with *E*. *muris* antigen of LMNCs from *E*. *muris*-primed mice secreted higher levels of IL-12 and IL-15 in comparison with IOE-primed LMNCs. In contrast, IOE infection that fails to induce memory-like NK cells or provide a protective recall response to *Ehrlichia* promoted the production of inflammasome-dependent, pro-inflammatory cytokines, including IL-1α, IL-1β, and IL-18 (data not shown), and the immunosuppressive IL-10. These data suggest that *E*. *muris* infection creates the optimal cytokine environment for supporting the generation of NK cells with adaptive memory features, which leads to an effective memory response.

Unlike IOE infection, primary *E*. *muris* infection causes persistent infection in B6 mice, despite the ability of the host to mount a strong cell-mediated immune response. Our recent studies demonstrated that persistent infection plays a role in the induction and maintenance of a high frequency of effector memory CD4^+^ T cells in *E*. *muris*-primed mice. However, the mechanism through which Ag persistence promotes memory T cell responses is not clear. The finding that hepatic NK cells expand and are activated, albeit at a lower frequency, in *E*. *muris*-primed mice at late time points after infection suggests that memory-like NK cells could be continuously activated by persistent *E*. *muris* infection. However, persistent infection does not appear to be critical for the survival of memory-like NK cells because NK cells transferred from the spleen and liver collected on day 21 from *E*. *muris-*primed mice into antigen–free hosts (*Rag2*^−/−^*Il2rg*^−/−^ mice) survived for a similar period as naïve NK cells. This finding suggests that *E*. *muris-*primed NK cells can survive in lymphopenic hosts and in the absence of persistent infection. More importantly, transferred memory-like NK cells into *Rag2*^−/−^*Il2rg*^−/−^ recipients maintained their protective functions because they were able to mediate a robust memory response against challenge with the same priming pathogen (*E*. *muris*). Our findings are consistent with the results obtained in other recent studies [42,47–50], which showed that infections with certain viruses, such as mouse cytomegalovirus (MCMV), induce memory-like Ly49H^+^ NK cells. In the present study, we did not examine specific markers expressed on memory-like NK cells induced by *E*. *muris* infection, however, the prolonged survival of *E*. *muris* primed NK cells and their ability to provide recall responses against *Ehrlichia* suggests that these NK cells contribute to memory response against *Ehrlichia*. Although we have not examined the antigen-specificity of the memory response, it is possible that *E*. *muris*-primed NK cells may acquire the adaptive feature of memory cells, similar to conventional T cells, a possibility that will be examined further in future studies. In conclusion, our study suggests that NK cells may possess attributes of innate and adaptive immunity and contribute to recall responses against obligate intracellular *Ehrlichia* that target the liver.

## Supporting Information

S1 Fig Anti-asialo-GM1 antibodies did not affect the number of T cells or NKT cells in *E*. *muris*/IOE-infected mice as measured by flow cytometry analysis of splenocytes on day 3 after secondary IOE infection. (TIF)

S2 Fig Anti-asialo-GM1 antibodies did not affect the number of T cells or NKT cells in *E*. *muris*/IOE-infected mice as measured by flow cytometry analysis of splenocytes on day 5 after secondary IOE infection. (TIF)
