# Supplementary figures and images for: NK Cell-Mediated Regulation of Protective Memory Responses against Intracellular Ehrlichial Pathogens
Source: PLoS One. 2016 Apr 19;11(4):e0153223. doi: 10.1371/journal.pone.0153223 (PMC4836677; doi:10.1371/journal.pone.0153223)

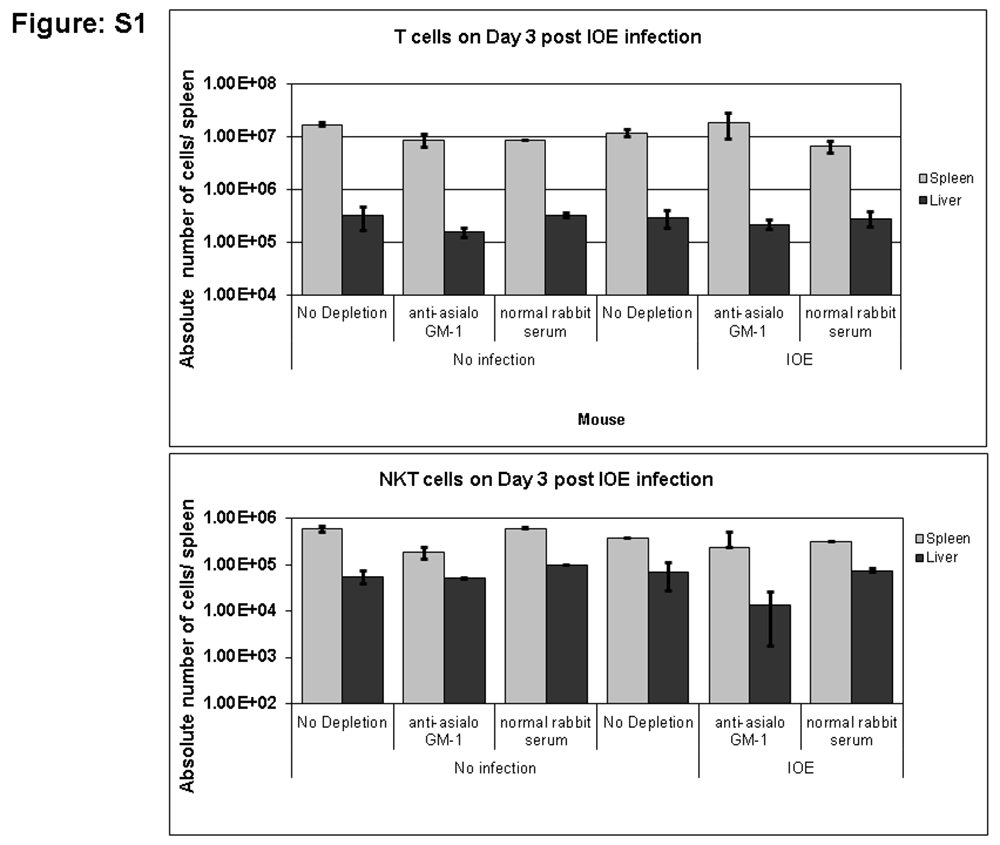

Supplement: S1 Fig — (TIF) [file pone.0153223.s001.tif]

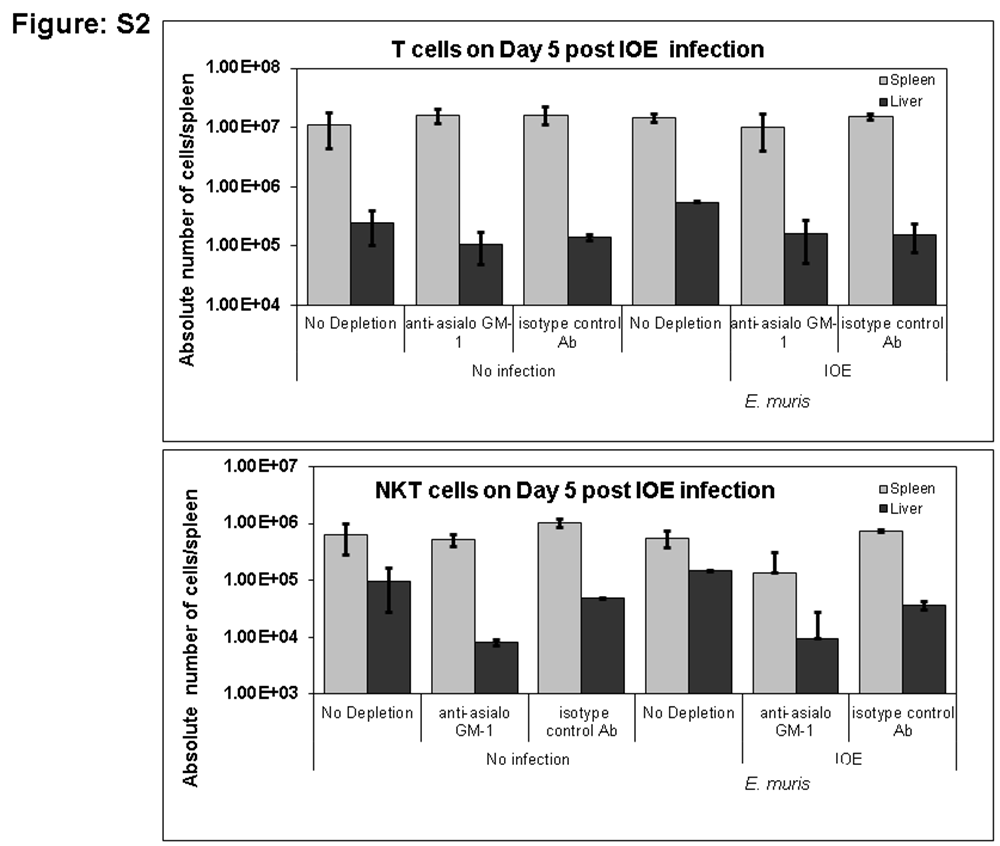

Supplement: S2 Fig — (TIF) [file pone.0153223.s002.tif]
